# Discretizing Three‐Dimensional Oxygen Gradients to Modulate and Investigate Cellular Processes

**DOI:** 10.1002/advs.202100190

**Published:** 2021-06-21

**Authors:** Michael R. Blatchley, Franklyn Hall, Dimitris Ntekoumes, Hyunwoo Cho, Vidur Kailash, Rafael Vazquez‐Duhalt, Sharon Gerecht

**Affiliations:** ^1^ Department of Biomedical Engineering Johns Hopkins University Baltimore MD 21218 USA; ^2^ Department of Chemical and Biomolecular Engineering Institute for NanoBioTechnology and Johns Hopkins Physical Sciences‐Oncology Center Johns Hopkins University Baltimore MD 21218 USA; ^3^ Department of Biophysics Johns Hopkins University Baltimore MD 21218 USA; ^4^ Department of Bionanotechnology Center for Nanosciences and Nanotechnology National Autonomous University of Mexico Ensenada Baja California 22800 Mexico; ^5^ Department of Materials Science and Engineering Johns Hopkins University Baltimore MD 21218 USA; ^6^ Department of Oncology Johns Hopkins University School of Medicine Baltimore MD 21205 USA

**Keywords:** cell survival, hydrogels, hypoxia, oxidative stress, vasculogenesis

## Abstract

With the increased realization of the effect of oxygen (O_2_) deprivation (hypoxia) on cellular processes, recent efforts have focused on the development of engineered systems to control O_2_ concentrations and establish biomimetic O_2_ gradients to study and manipulate cellular behavior. Nonetheless, O_2_ gradients present in 3D engineered platforms result in diverse cell behavior across the O_2_ gradient, making it difficult to identify and study O_2_ sensitive signaling pathways. Using a layer‐by‐layer assembled O_2_‐controllable hydrogel, the authors precisely control O_2_ concentrations and study uniform cell behavior in discretized O_2_ gradients, then recapitulate the dynamics of cluster‐based vasculogenesis, one mechanism for neovessel formation, and show distinctive gene expression patterns remarkably correlate to O_2_ concentrations. Using RNA sequencing, it is found that time‐dependent regulation of cyclic adenosine monophosphate signaling enables cell survival and clustering in the high stress microenvironments. Various extracellular matrix modulators orchestrate hypoxia‐driven endothelial cell clustering. Finally, clustering is facilitated by regulators of cell–cell interactions, mainly vascular cell adhesion molecule 1. Taken together, novel regulators of hypoxic cluster‐based vasculogenesis are identified, and evidence for the utility of a unique platform is provided to study dynamic cellular responses to 3D hypoxic environments, with broad applicability in development, regeneration, and disease.

## Graphical abstract

An approach to discretize oxygen gradients within hydrogels is presented to enable the study of 3D cell behavior in biomimetic microenvironments without the need for technical expertise or specialized equipment. The platform is used to study vasculogenesis, and new mechanistic details are uncovered, highlighting the utility of this system for the investigation of the role of oxygen in biological phenomena.

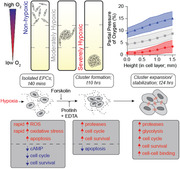

## 1 Introduction

The level of oxygen (O_2_) in tissue microenvironments plays a key role throughout development, as well as in adult tissue and organ homeostasis, regeneration, and disease.^[^ ^1^, ^2^, ^3^ ^]^ A particular focus has been on the role of low levels of O_2_, or hypoxia (<5% O_2_), in cardiovascular disease and cancer, making it a hallmark of the microenvironment in both diseases.^[^ ^4^ ^]^ Indeed, hypoxia has proven a potent regulator of thousands of genes governing numerous biological pathways in both clinical presentations.^[^ ^5^, ^6^ ^]^ As such, uncovering the details describing the mechanisms by which hypoxia can influence cancer progression and cardiovascular disease and regeneration has become subject of intense study spanning fundamental biology to translational research. Interestingly, manipulation of neovessel formation is foundational to promising treatment approaches for both diseases, where therapies for ischemic cardiovascular disease seek to promote neovessel formation and infiltration, while those developed to treat cancer aim to inhibit tumor vessel infiltration (also known as angiogenesis), and associated metastasis.

Accurately studying neovessel formation in vitro requires a 3D platform that can mimic the native vascular regenerative microenvironment.^[^ ^7^ ^]^ Hydrogels, both naturally derived and synthetic, can act as mimics of the extracellular matrix (ECM) and can provide the means to manipulate a variety of properties, such as stiffness, integrin binding, and degradability to construct engineered vascular networks. These hydrogels have helped uncover many important regulators of angiogenesis and vasculogenesis, but most of these experiments have been conducted in atmospheric (21%) O_2_, neglecting to incorporate a key property of the angiogenic microenvironment.

Established approaches for studying hypoxia most often only study a user‐determined level of oxygen (e.g., 1% O_2_ or 5% O_2_), and require the use of either hypoxia chambers or specialized hypoxia glove boxes that are flushed with mixed gases to achieve the desired level of O_2_. These systems are unable to study cell behavior in more biomimetic O_2_ gradients. Several groups have developed microfluidics‐based systems or intricately designed 3D rollable biomaterials to generate O_2_ gradients, but both of these platforms require technical expertise to fabricate and use.^[^ ^8^, ^9^ ^]^ To combat this multifaceted array of limitations, we have previously designed gelatin‐based O_2_‐controllable hydrogels to mimic the hypoxic vascular regenerative microenvironment.^[^ ^10^, ^11^, ^12^ ^]^ Importantly, these hydrogels do not require specialized equipment or technical expertise to generate biomimetic O_2_ gradients, and all experiments can be conducted in standard cell culture incubators.

In our previous works, we studied cell behavior in hypoxic gradient hydrogels, and compared the behavior of those cells to cells in nonhypoxic hydrogels.^[^ ^10^, ^11^, ^12^, ^13^, ^14^ ^]^ While these studies allowed for accurate recapitulation of new blood vessel formation and cluster‐based vascular morphogenesis in hypoxic conditions, non‐uniform cell behavior resulted from the presence of the O_2_ gradient in hypoxic conditions. We have found that the inherent diversity in cell responses across the O_2_ gradient impacted our ability to probe the details of the molecular signaling involved. Specifically, the use of conventional molecular biology techniques (e.g., qPCR, Western blot) resulted in readouts representing averages of gene and protein expression from cells in different local environments. As a result, differences between hypoxic and nonhypoxic conditions were difficult to accurately gauge, and some important differences in gene expression were likely masked, particularly at early time points where differences in gene expression are less profound.

Here, we developed an approach to study uniform cell behavior in 3D hypoxic microenvironments by discretizing O_2_ gradients, thus enabling in‐depth analysis of the molecular mechanisms guiding cluster formation. Using RNA‐sequencing, we found a distinctive pattern of global gene expression at discretized O_2_ concentrations, allowing us to expand upon previous findings of the role of reactive oxygen species (ROS) and oxidative stress, protease production, and cell–cell interactions in vascular cell clustering. Importantly, using the new method, we identified time‐dependent regulation of cyclic adenosine monophosphate (cAMP) signaling, cell survival pathways, inhibition of apoptosis, cell cycle upregulation, and increased carbohydrate metabolism as critical for vascular cluster formation.

## 2 Results and Discussion

### 2.1 Development and Characterization of Layered O_2_‐Controllable Hydrogels

**Figure 1:**
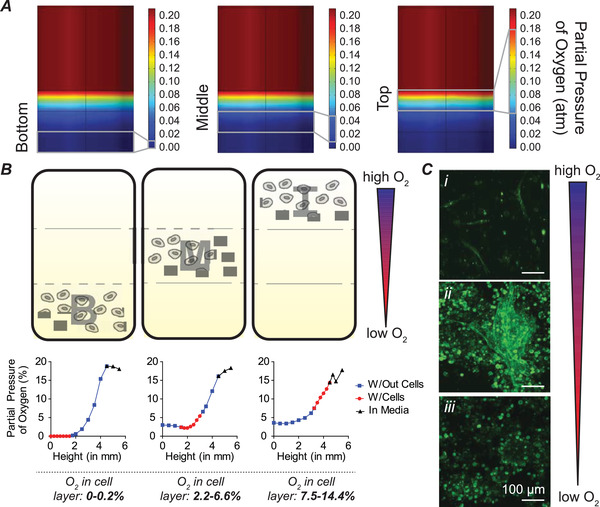
Discretized O_2_‐gradients facilitate study of cell behavior in refined 3D microenvironments. A) Computational model of layered hydrogels showing bottom (severely hypoxic), middle (moderately hypoxic), and top (nonhypoxic) layers. B) Cells were encapsulated in bottom (B; severely hypoxic), middle (M; moderately hypoxic), and top (T; nonhypoxic) layers. Representative O_2_ measurements are shown. C) Distinct cell morphology was observed in each layer. Cells in the T layer exhibit single cell vasculogenesis (i); cells in the M layer exhibit cluster formation and vascular sprouting from clusters (ii); and cells in the B layer exhibit cluster formation (iii). Images were captured at D3 (72 h) after cell encapsulation. All scale bars: 100 µm.

In previous works, we designed O_2_‐controllable hydrogels using phenol‐conjugated gelatin enzymatically cross‐linked by laccase, with resultant O_2_‐consumption during the cross‐linking reaction (Figure S1A, Supporting Information).^[^ ^10^, ^12^ ^]^ This reaction, alongside O_2_ consumption by the encapsulated cells, resulted in hypoxic gradient hydrogels ranging from <1–21%, with gradient conditions sustained over the experimental time course of days, as dependent on cell type and cell concentration.^[^ ^13^, ^14^ ^]^ To refine and advance our hydrogel design, we speculated that creating distinct layers of O_2_‐controllable hydrogels, where cells are encapsulated in a specific layer, would create a defined region with a predictable and controllable O_2_ concentration, where we could study cell response to discretized O_2_ gradients. We developed a computational model to better understand how to tune our hydrogel geometry to yield distinct O_2_ gradients to allow precise study of cellular responses to variable levels of O_2_ (see “Experimental Section”). We determined that a layer‐by‐layer approach would allow us to generate narrow ranges of hypoxic O_2_ concentrations in a highly defined manner, thus creating severely hypoxic, moderately hypoxic, and nonhypoxic conditions using three layers (**Figure** **1**A), or hypoxic and nonhypoxic conditions using two layers (Figure S2, Supporting Information). To test this experimentally, we assembled the hydrogels in a layer‐by‐layer fashion and, during fabrication, encapsulated endothelial colony forming cells (ECFCs; a subtype of EPCs) within the three distinct layers in the O_2_‐controllable hydrogels, each corresponding to different O_2_ levels. O_2_ measurements were recorded using needle‐type O_2_ sensors. In the bottom layer, cells were exposed to severe hypoxia (0–0.2% O_2_), in the middle layer, cells were exposed to moderate hypoxia (2.2–6.6% O_2_), and in the top layer, cells were exposed to nonhypoxic conditions (7.5–14.4% O_2_) (Figure 1B and Figure S3, Supporting Information). In each layer, a distinct cell morphology was observed. In the bottom (severely hypoxic) layer, ECFC clusters formed, with no sprouting (Figure 1C[i]). In the middle (moderately hypoxic) layer, ECFC clusters formed, with extensive sprouting from clusters (Figure 1C[ii]), and in the top (nonhypoxic) layer, vascular sprouting resembling a different mechanism for new blood vessel formation, single‐cell‐based vasculogenesis, was observed (Figure 1C[iii]).^[^ ^15^ ^]^ Importantly, because these hydrogels are cross‐linked enzymatically, and one reaction is not fully completed upon the sequential addition of each subsequent layer, the timing of the layer‐by‐layer fabrication of our hydrogels resulted in a final structure with uniform, fused layers.

### 2.2 Layered Hydrogels Facilitate Study of Uniform Cell Behavior and Dynamics

**Figure 2:**
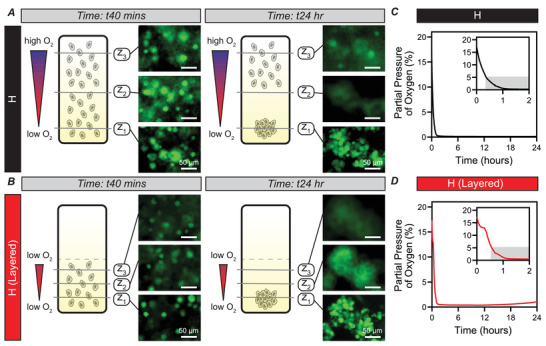
Layered‐hypoxic hydrogels enable uniform ECFC cluster behavior, and match conventional hypoxic hydrogel cluster formation kinetics and O_2_. A,B) ECFCs were encapsulated in conventional hypoxic hydrogels (A) and in the layered‐hypoxic hydrogels (B). 40 min after encapsulation, uniform cell seeding was confirmed in both conditions, as measured by images taken at three distinct z‐planes (z1 [lowest] → z3 [highest]). After 24 h in culture, clusters formed in both hydrogels. In the conventional hypoxic hydrogel, clusters were in focus at z1, with no cells in focus at z2 but cells in single cell morphology at z3, indicating non‐uniform cell behavior. In the layered‐hypoxic hydrogels, uniform cell behavior was observed, with all cells in focus at z1, and no cells in focus at z2 or z3. C,D) O_2_ measurements show rapid exposure to hypoxia (<5% O_2_, grayed area in inset) in both conditions and exposure to hypoxia over the entire 24 h culture period. All scale bars: 50 µm.

Cluster‐based vasculogenesis from circulating endothelial progenitor cells has been reported under hypoxic conditions.^[^ ^13^, ^16^, ^17^ ^]^ Thus, we sought to focus on uncovering the regulatory mechanism governing this clustering process. This process is rapid, with cluster formation by 10 h, and cluster expansion and stabilization by 24 h. As such, we limited the studies throughout the remainder of the manuscript to 24 h. To examine whether our approach would allow us to study uniform cluster formation in a 3D hypoxic environment with discretized O_2_ gradients, we used the two‐layer approach to allow robust cell studies. We analyzed ECFCs encapsulated in the bottom layer and ECFCs encapsulated in our conventional hypoxic hydrogels. In both conditions, we observed uniform cell seeding throughout the z‐plane at an early time point, 40 min after encapsulation (**Figure** **2**A,B; left). After 24 h in culture, clustering of ECFCs was observed in both conditions (Figure 2A,B; right). However, in the layered hypoxic condition (Figure 2B; right), nearly all cells participated in cluster formation (at the lowest z‐plane, z1), with no cells in focus in the higher z‐planes (z2 and z3). In the conventional hypoxic hydrogel (Figure 2A; right), clusters appeared at the lowest z‐plane (z1), no cells were in focus at the intermediate z‐plane (z2), and cells appeared in isolation at the highest z‐plane (z3), revealing heterogeneity in cell behavior.

We also confirmed the kinetics of cluster formation in the layered‐hypoxic hydrogels matched cluster formation kinetics in conventional hypoxic hydrogels. In both conditions, cells were seeded uniformly in a single cell morphology, then clusters were formed by 10 h in culture, and ultimately expanded in terms of number of cells in clusters by 24 h in culture (Figure S1B, Supporting Information). Accordingly, measurements of O_2_ at the bottom of the hydrogel matched along the entirety of the culture period, with rapid exposure to hypoxia (<5% O_2_) in both conditions, and maintenance of hypoxia throughout the 24 h culture period (Figure 2C,D). Hence, culture of ECFCs in the layered hypoxic condition resulted in uniform cell behavior matching the cluster formation kinetics of ECFCs cultured in conventional hypoxic gradient hydrogels used in previous studies, highlighting the utility of this system for use in analyzing differential gene expression of cells cultured in 3D biomimetic hypoxic conditions.

### 2.3 Global View of RNA Sequencing of Layered‐Hypoxic and Nonhypoxic Hydrogels

**Figure 3:**
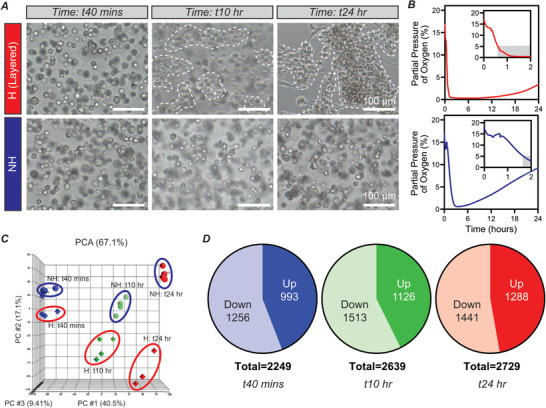
Differential gene expression between ECFCs encapsulated in layered‐hypoxic and nonhypoxic hydrogels. A) ECFCs encapsulated in layered‐hypoxic hydrogels exhibited cluster formation, while cells in nonhypoxic hydrogels remained isolated as single cells. B) O_2_ measurements of the layered‐hypoxic hydrogels indicated rapid and sustained exposure to hypoxia. O_2_ measurements at the bottom of nonhypoxic hydrogels indicated delayed and transient exposure to hypoxia. C) PCA identifies three PCs contribute to 67.1% of the variance in the data. Biological replicates for each condition (hypoxic vs nonhypoxic) cluster together at each time points. Outliers, as identified by statistical analysis, were omitted from subsequent analysis. *n* = 3–4 for each condition at each time point. D) Statistically significantly differentially expressed genes in hypoxic versus nonhypoxic conditions at each time point, as identified by 1‐way ANOVA. All scale bars: 100 µm.

Following confirmation of uniform cell behavior and accurate mimicry of cluster formation kinetics and O_2_ between layered and conventional hypoxic hydrogels, we next focused on identifying new signaling regulating hypoxic cluster formation. Toward this, we performed RNA sequencing on cells encapsulated in layered hypoxic hydrogels (bottom layer; hereafter referred to as layered‐hypoxic hydrogels or H) and cells encapsulated in nonhypoxic hydrogels (NH), which do not exhibit cluster formation (**Figure** **3**A). Non‐invasive measurements of O_2_ at the bottom of the hydrogel revealed rapid exposure to hypoxic levels of O_2_ that were maintained over the culture period (Figure 3B). Interestingly, cells in nonhypoxic hydrogels experienced delayed and transient exposure to hypoxic conditions, indicating the importance of rapid exposure to hypoxia in cluster formation (Figure 3B). At predetermined time points, 40 min after encapsulation, 10 h after encapsulation, and 24 h after encapsulation, we collected and purified RNA for RNA sequencing. Principle component analysis (PCA) confirmed clustering of experimental conditions at each time point, verifying differential gene expression between the two conditions at all three time points (Figure 3C). A global view of the sequencing data clearly identifies thousands of statistically significantly differentially expressed genes between cells exposed to hypoxic or nonhypoxic conditions at each time point (Figure 3D). Using Ingenuity Pathway Analysis (IPA), we identified several other governing pathways and genes guiding ECFC cluster formation upon rapid exposure to biomimetic hypoxic conditions in 3D engineered microenvironments (Figures S4–S9, Supporting Information).

### 2.4 Genes Associated with Oxidative Stress and cAMP Signaling Regulate Cluster Formation upon Rapid Exposure to Hypoxic Conditions

**Figure 4:**
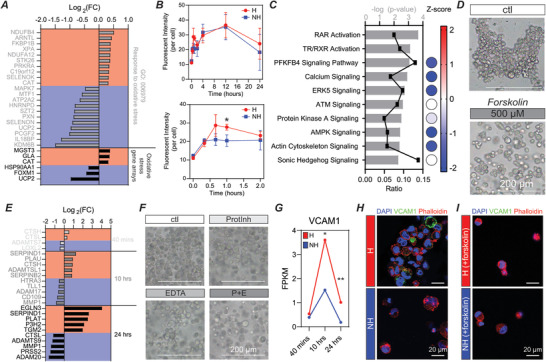
Analysis and confirmation of RNA‐sequencing data reveals the role of ROS and oxidative stress, cAMP signaling, matrix degradation by proteases, and the role of VCAM‐1 in cluster formation and stabilization. A) Top differentially expressed genes in the Gene Ontology: 0006979 pathway and in a set of oxidative stress associated genes. All genes are statistically significantly differentially expressed and have a value of >ǀ2*σ*ǀ based on standard deviation analysis at the 40 min time point. Red indicates upregulation. Blue indicates downregulation. B) Quantification of CellROX reveals significant upregulation of ROS, and associated oxidative stress, at early time points in layered hypoxic conditions compared to nonhypoxic conditions. *n* = 3 independent experiments per condition. Graphical data are reported as mean ± SD. *p < 0.05. C) Curated IPA pathways at the 40 min time point. All pathways have statistically significant overlap. Ratios indicate the number of genes in our dataset compared to the total number of genes in each pathway. Activation Z‐score indicates activation (+ value) or deactivation (− value). D) Forskolin (cAMP agonist) inhibits cluster formation in hypoxic conditions. Scale bars: 200 µm. E) Top differentially expressed genes in the Matrisome Project gene set ECM regulators. All genes are statistically significantly differentially expressed and have a value of >ǀ2*σ*ǀ based on standard deviation analysis at the associated time point. Red indicates upregulation. Blue indicates downregulation. F) ProtInh and EDTA both partially inhibit cluster formation when used in isolation, but result in substantial reduction in cluster formation when used as a combination inhibitor (P + E). Scale bars: 200 µm. G) FPKM of VCAM1 over the experimental time course. H) VCAM1 expression in hypoxic and nonhypoxic conditions at the 24 h time point. I) VCAM expression in forskolin treated hypoxic and nonhypoxic conditions at the 24 h time point. Scale bars (H,I): 20 µm. *p < 0.05, **p < 0.01, ***p < 0.001, ****p < 0.0001.

Rapid upregulation of ROS facilitates cluster formation in conventional hypoxic hydrogels.^[^ ^13^ ^]^ Armed with this knowledge, we analyzed gene sets related to ROS and oxidative stress defined by commercially available gene arrays (see Experimental Section), as well as Gene Ontology and Hallmark Gene Sets available through the Gene Set Enrichment Analysis (GSEA) database.^[^ ^18^, ^19^ ^]^ Curating this data, we identified numerous differentially expressed genes over the experimental time course (Figures S10 and S11, Supporting Information). Because of the role of ROS and oxidative stress at early time points, we focused our analysis on the 40 min time point. Here, we looked at Gene Ontology: 0006979, response to oxidative stress, as well as a set of 95 genes related to oxidative stress from a gene array of curated genes related to oxidative stress. Interestingly, these two defined gene sets, nominally defined to cover identical processes, contained sets of unique genes, with only 70 overlapping (Figure S11A, Supporting Information). At the 40 min time point, we identified the top differentially expressed genes in the Gene Ontology pathway as well as the gene array set that were statistically significantly different and that met the threshold of our standard deviation analysis (**Figure** **4**A, see Experimental Section). Further, we investigated genes in the Hallmark Reactive Oxygen Species Pathway in GSEA and identified additional differentially expressed genes (Figure S12A,B, Supporting Information). Many of the genes included in our analysis follow the same trend, with upregulation in H versus NH conditions. However, it is not unexpected to see some genes within our analyzed gene sets, such as ABCC1 and SCAF4 following the opposite trend, as these genes may be involved in numerous signaling pathways. To confirm the role of ROS and oxidative stress in the layered‐hypoxic hydrogels, we co‐encapsulated CellROX, which fluoresces upon oxidation with ROS, to identify oxidative stress in live cells. While cells cultured in layered‐hypoxic hydrogels and nonhypoxic hydrogels exhibit CellROX+ fluorescence (Figure 4B and Figure S13, Supporting Information), there was a statistically significant increase in fluorescence at early time points in layered hypoxic conditions compared to nonhypoxic conditions, confirming the role of ROS in driving cluster formation (Figure 4B). Taken together, these analyses confirm our findings of the critical importance of rapid ROS production guiding cluster formation

IPA at the 40 min time point guided our analysis further to understand overlap between the differentially expressed genes in our dataset and existing defined pathways. Many of the top pathways identified were related to oxidative stress (Figure 4C). Interestingly, most of these pathways have a negative Z‐score, indicating they are deactivated at this time point. Looking specifically at the genes differentially expressed at the 40 min time point and overlapping with these top pathways (Figure S12C,D, Supporting Information), we observed additional genes not identified in the oxidative stress or ROS pathways. Many of the identified pathways, including calcium signaling, protein kinase A signaling, AMPK signaling, and Sonic Hedgehog signaling, are related to cAMP signaling. Because most of these genes and pathways were downregulated, we sought to activate cAMP to inhibit cluster formation. Here, we used a cAMP agonist, forskolin, and saw inhibition of cluster formation, confirming the role of cAMP signaling in the formation of ECFC clusters when exposed to conditions leading to oxidative stress (Figure 4D).

### 2.5 Genes Associated with Matrix Remodeling and Cell–Cell Interactions Are Differentially Expressed over the Experimental Time Course

Our previous work established the importance of matrix degradation and remodeling in facilitating cluster formation, with upregulation of proteases resulting in rapid matrix degradation, which facilitated cell clustering through passive migration to void spaces cleared by proteolysis. Here, we have also confirmed a significant reduction in shear modulus (G′) at the onset of cluster formation (10 h) (Figure S14A, Supporting Information), further supporting that rapid matrix degradation drives cluster formation. To deepen our understanding of the role of matrix degradation, we utilized data compiled by the Matrisome Project, specifically analyzing ECM regulators defined as “genes encoding enzymes and their regulators involved in the remodeling of the extracellular matrix,”^[^ ^20^ ^]^ and saw differential expression of numerous genes throughout the experimental time course (Figure 4E and Figure S14B,C, Supporting Information). Interestingly, we previously analyzed an array of soluble proteases and observed many proteases were present in both hypoxic and nonhypoxic conditions, but we only saw significant upregulation of MMP‐1 in hypoxic conditions. Even with a broad spectrum MMP inhibitor (GM6001), we did not see complete inhibition of cluster formation.^[^ ^13^ ^]^ This result is not surprising in light of the vast array of genes encoding proteins responsible for matrix remodeling that were upregulated in hypoxic conditions at the RNA level (Figure 4E and Figure S14B,C, Supporting Information). To confirm our hypothesis that numerous genes are involved in matrix degradation that leads to cluster formation, we knocked down MMP1 with siRNA, and observed no inhibition of cluster formation compared to the scrambled control (Figure S15A, Supporting Information). In fact, MMP1 was significantly downregulated at later time points in hypoxic versus nonhypoxic conditions, while both conditions had high read counts, indicating that MMP1 is being transcribed in both over the time course (Figure S15A, Supporting Information). We further tested inhibition of CTSL, which has been shown to play a crucial role in EPC‐mediated matrix degradation.^[^ ^21^ ^]^ Concentrations of CTSL inhibitor (Z‐FF‐FMK) ranging from 10 to 500 µm did not inhibit cluster formation (Figure S15B, Supporting Information). CTSL RNA levels increased over time in both conditions, with significant upregulation at 40 min in hypoxic versus nonhypoxic conditions and the opposite trend at later time points. We then utilized a protease and phosphatase inhibitor cocktail (ProtInh) to broadly inhibit proteases, including serine and cysteine proteases, and saw partial inhibition of cluster formation (Figure 4F). We saw similar results with metalloprotease inhibitor EDTA (Figure 4F), but saw marked reduction in cluster formation with a combination inhibitory cocktail containing both ProtInh and EDTA (Figure 4F, P + E). Single factor protease inhibition is not sufficient to inhibit cluster formation, but rather requires broad protease inhibition. These results provide insights into the potent nature of hypoxia as an inductive cue regulating cluster formation through upregulation of numerous ECM regulator genes, some of which directly impact matrix remodeling through enzymatic degradation, and some of which have an indirect effect by activating or inactivating matrix remodeling proteins.

In addition to proteolytic degradation, ECM biosynthesis and newly deposited matrix components are important in vascular development and regeneration.^[^ ^22^, ^23^, ^24^ ^]^ As such, we analyzed genes encoding for previously identified ECM proteins important in angiogenesis and vascular development. Here, we identified a number of genes characterized as “pro‐angiogenic,” which were upregulated over time in both conditions, but most often with higher expression in NH compared to H conditions (Figure S16A, Supporting Information). Additionally, several “anti‐angiogenic” genes were downregulated over time in both conditions (Figure S16B, Supporting Information). These trends confirm the importance of cellular tuning of the local environment to a pro‐regenerative state to facilitate the formation of new blood vessels by both classical single‐cell‐based vasculogenesis (NH) and cluster‐based vasculogenesis (H). Differential regulation of other ECM genes warrants further investigation, specifically with regard to their role in cluster‐based vasculogenesis (Figure S16C, Supporting Information).

We previously identified cell–cell interactions through VE‐cad (CDH5), ICAM1, and ITGB2 as regulators of cluster stabilization at the protein level through localization at the cell–cell junction. Here, we observed decreasing CDH5 in both conditions, with both maintaining relatively high read counts over the experimental time course, and increases in ICAM1 over time in both conditions with nonhypoxic conditions yielding higher expression at the 24 h time point (Figure S15C, Supporting Information). ITGB2 had low read counts in both conditions (Figure S15C, Supporting Information). These results likely indicate that rather than increasing expression of these proteins in hypoxic conditions, cells localize these proteins to the cell–cell junction to stabilize ECFC clusters. Looking more in‐depth into regulators of cell–cell interactions, IPA analysis revealed significant overlap and activation of the biological function “binding of endothelial cell lines” at the 24 h time point (Figure S15D, Supporting Information). The overlapping genes in this process are unique from our previously identified proteins, with the most significant increase in vascular cell adhesion molecule 1 (VCAM1) (Figure S15E, Supporting Information), which increased at 10 h then decreased at 24 h, but importantly at both time points VCAM1 expression was significantly increased under hypoxic versus nonhypoxic conditions (Figure 4G). The role of VCAM1 was further supported by immunostaining at the 24 h time point, revealing increased protein‐level expression of VCAM1 in hypoxic versus nonhypoxic conditions (Figure 4H). When cluster formation is inhibited by treatment with forskolin, VCAM1 expression in hypoxic conditions is also inhibited, suggesting that VCAM1 is particularly important within ECFC clusters, and not simply upregulated by hypoxia in our system (Figure 4I).

Use of RNA‐sequencing to study differential gene expression of uniform cell behavior in complex, 3D environments confirmed our previous findings, as well as provided critical utility in identification of additional genes regulating cluster formation and cluster stabilization, including an array of proteases and VCAM1, which is likely critical toward regulating cluster stabilization and numerous other signaling pathways critical to new blood vessel formation.^[^ ^25^, ^26^ ^]^ Importantly, we also showed that many genes are involved in cluster formation, and single factor inhibition fails to abrogate cluster formation, highlighting the requirement for multifactorial inhibition or regulation of pathways involving numerous genes critical to the early stages of the cluster formation process (e.g., cAMP signaling) to limit cluster‐based vasculogenesis.

### 2.6 Cell Survival, Apoptosis, and Cell Cycle Progression Are Differentially Regulated Concomitant with Cluster Formation

**Figure 5:**
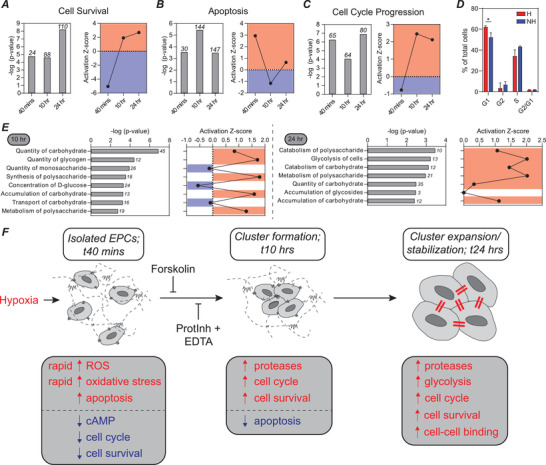
Cell survival, apoptosis, cell cycle progression, and metabolism are differentially regulated as ECFC clusters form. A) There is a statistically significant overlap in the genes from our dataset and the set of genes regulating cell survival. Bars are the −log (*p*‐value), with the number of relevant genes listed above each bar. The activation Z‐score predicts that cell survival is downregulated at the 40 min time point and upregulated at 10 and 24 h. B) Statistically significant overlap between genes in our dataset and those associated with apoptosis. This set of genes is initially upregulated (40 min), then downregulated (10 h), and then upregulated again (24 h). C) Statistically significant overlap between genes in our dataset and those associated with cell cycle progression. This set of genes is initially downregulated (40 min), then upregulated at 10 and 24 h. D) Flow cytometry analysis of cell cycle with propidium iodide staining. E) Many biological functions associated with carbohydrate metabolism are significantly upregulated at the 10 and 24 h time points. *p < 0.05, **p < 0.01, ***p < 0.001, ****p < 0.0001. F) Proposed mechanism for cluster formation and stabilization. Hypoxia leads to upregulation of ROS and oxidative stress. In response to ROS, genes encoding antioxidants are upregulated to protect cells from oxidative stress. At early time points, pathways associated with cAMP signaling are downregulated, and a cAMP agonist, forskolin, inhibits cluster formation. Proteases are then upregulated to degrade the matrix and clusters form. Broad spectrum, multitarget protease inhibitors block cluster formation. Increases in cell survival and cell cycle progression, as well as decreases in apoptosis are associated with cluster formation. Upregulation of carbohydrate metabolism and cell–cell interactions occur at later time points.

IPA analysis provided additional insight into new pathways involved in cluster formation. At the 40 min time point, genes associated with cell survival were downregulated in hypoxic conditions, but genes associated with this pathway were then upregulated at the 10 and 24 h time points (**Figure** **5**A and Figure S17A,B, Supporting Information). We predict this switch is due to the initial microenvironment causing an upregulation in cell stress and an associated reduction in cell survival signaling, but then cluster formation at later time points facilitates an upregulation in cell survival signaling to overcome the high stress microenvironment.

Analyzing a related pathway, apoptosis, we saw a nearly opposite trend (Figure 5B). At the early time point, genes associated with apoptosis were upregulated, and then as clusters form, apoptosis was downregulated. The 24 h time point is slightly puzzling, as cell survival was increased, but some apoptosis is required for vascular lumen formation, so apoptosis may be a requirement for cluster‐based vascular network lumen formation. This is an interesting line of inquiry outside the scope of the work presented here.

Genes associated with cell cycle progression follow a similar trend to those associated with cell survival, with many genes upregulated at the 10 and 24 h time points (Figure 5C and Figure S17C, Supporting Information). We confirmed this finding by analyzing cell cycle with flow cytometry (Figure 5D), and saw that a variety of biological functions were activated to contribute to nearly all stages of the cell cycle at both the 10 and 24 h time points, perhaps most notably, the pathway defined by genes associated with the G1 phase, which was statistically significantly upregulated by flow cytometry analysis, was a top hit at both 10 and 24 h (Figure S17D,E, Supporting Information). In summary, when exposed to a hypoxic environment, ECFCs form multicellular clusters to enhance cell survival, reduce apoptosis, and progress through the cell cycle.

### 2.7 Carbohydrate Metabolism Is Upregulated in Hypoxic Conditions

ECs are known to be highly glycolytic. The role of metabolism in angiogenesis has become a topic of widespread interest, pioneered by the Carmeliet group, where EC metabolism has been established as a driver rather than a bystander in angiogenesis.^[^ ^27^, ^28^ ^]^ Analysis of biological functions associated with carbohydrate metabolism revealed enrichment and activation at both the 10 and 24 h time points (Figure 5E). Assessment of the specific genes associated with the “metabolism of polysaccharide” and “glycolysis” functions revealed numerous differentially expressed genes (Figure S18A–D, Supporting Information). HK2 represents a potential target gene because of its significant differential expression, as well as its upstream role in a rate‐limiting step in glucose metabolism (Figure S18D, Supporting Information).^[^ ^29^ ^]^ These results indicate that ECFCs within clusters adapt to the low O_2_ environment by altering their metabolism. Because of the significant changes in metabolic pathways at later time points in our analysis, we posit that blocking or activating this shift in metabolism is unlikely to influence cluster formation, but it may be important at later stages of cluster‐based vasculogenesis, especially when cells begin to sprout from clusters, and should be the focus of future studies.

## 3 Conclusion

Utilizing layered hydrogels with discretized O_2_ gradients, we were able to design a 3D hydrogel platform that facilitated study of uniform ECFC cluster formation. This novel design accurately recapitulated the cluster formation mechanism we have previously defined, and allowed for in‐depth analysis of the molecular regulators of cluster formation using RNA sequencing and pathway analysis. Further, the system facilitated rapid exposure to low O_2_, which closely mimics the biological environment to which EPCs home in vivo. Using the layered hydrogel platform, we have delineated a detailed mechanism for cluster formation, as shown in Figure 5F. Specifically, we confirmed the role of early upregulation of oxidative stress and ROS, and identified novel regulators of the process, including cAMP signaling. We then confirmed upregulation of numerous ECM regulators, which we hypothesize have direct effects on matrix degradation, and indirect effects on matrix degradation through coordinated activation of other matrix‐degrading proteases to facilitate cluster formation. Using broad spectrum protease inhibitors, but not single factor inhibitors, we successfully blocked cluster formation. We then confirmed the role of cell–cell interactions in cluster stabilization and identified VCAM1 as an additional regulator.

IPA identified new pathways and potential target genes involved in cluster formation, including cell survival, anti‐apoptosis, and cell cycle progression. Finally, upregulation of carbohydrate metabolism was observed at later time points. It is unknown whether alterations to carbohydrate metabolism influence cluster formation, but we predict they will impact vascular sprouting from clusters at time points outside the scope of the current work.

Overall, in this work, we have established a novel cell culture platform to investigate complex processes, such as hypoxia‐driven cluster‐based vasculogenesis, in a highly biomimetic in vitro microenvironment. Using this platform, we have provided a detailed analysis of the changes in gene expression over the time course in which ECFC clusters form, including several previously unknown signaling pathways involved in cluster formation and stabilization, leading to a detailed mechanism for cluster formation. Future studies could focus on uncovering the precise role of each of the signaling pathways identified in the current studies.

On a technical level, this study lays the groundwork for expanded utilization of O_2_‐controllable hydrogels, by facilitating in‐depth study of 3D cell behavior in discretized O_2_ gradients amenable to use in standard cell culture incubators. We anticipate that the utility of these layered O_2_‐controllable hydrogels will expand beyond the study of vasculogenesis. One potential area of interest is cancer biology, where this system could be used to study how hypoxia modulates immune cell migration in the tumor microenvironment. For our studies, we have specifically optimized the hydrogel properties for vasculogenesis, and thus, to expand the functionality of these hydrogel further, properties must be tuned in an application‐specific manner. For example, as the use of gelatin limits the tunability of hydrogels, in terms of the range of achievable changes to matrix mechanics, integrin binding, and degradability, the use of poly (ethylene glycol),^[^ ^30^ ^]^ dextran,^[^ ^11^ ^]^ or hyaluronic acid^[^ ^31^ ^]^ could be considered to enable a higher degree of tunability.

Biologically, we revealed that ECFCs enhance their survival in high stress microenvironments by forming cell clusters. Conceptually, in future works this hydrogel platform could serve as a delivery tool for O_2_‐responsive cells, where controlled hypoxia could facilitate clustering post‐implantation to guide regenerative cell therapies by enhancing cell survival in vivo. In sum, our O_2_‐controllable hydrogels with discretized gradients could serve as a tool to study regulators of hypoxia‐driven cellular dynamics in a highly biomimetic, 3D setting, with potential utility as a delivery system to enhance cell survival for cell‐based therapies.

## 4 Experimental Section

### Materials

Gelatin (Gtn, from porcine skin gel strength 300, Type A; G2500), trans‐ferulic acid (FA; 128708), *N*‐(3‐dimethylaminopropyl)‐*N*′‐ethylcarbodiimide hydrochloride (EDC; E6383), *N*‐hydroxysuccinimide (NHS; 56480), dimethyl sulfoxide (DMSO; 276855), cathepsin L inhibitor I (Z‐FF‐FMK; 219421), and bovine serum albumin (BSA; A3059), were purchased from Sigma‐Aldrich (St. Louis, MO) and used as obtained without purification. Laccase (976 U mL^−1^ from *Coriolopsis gallica*) was provided by collaborator Dr. Rafael Vazquez‐Duhalt, Centro de Nanociencias y Nanotecnología UNAM. Microbial transglutaminase (mTG) (Activa‐TI) was obtained from Ajinomoto Inc. or mTG (TI formula) from Moo Gloo. Dulbecco's phosphate‐buffered saline (DPBS; 14190250), trypsin‐EDTA 0.05% (25300120), Halt Protease and phosphatase inhibitor cocktail (ProtInh) (100×) and EDTA (78440), Trizol reagent (15596018), Molecular Probes CellROX Green Reagent (C10444), RNase A, DNase and protease‐free (RNase A, 10 mg/mL; FEREN0531), formaldehyde 37% by weight (formaldehyde; F79), Alexa Fluor 546 phalloidin (phalloidin; A22283), Goat anti‐Mouse Secondary Antibody Alexa Flour (ms488; A11001), Antibody diluent (003218), and Propidium Iodide (PI, 1.0 mg mL^−1^; P3566) were all purchased from Thermo Fisher Scientific (Waltham, MA). Human VCAM‐1/CD106 antibody (VCAM‐1; BBA5) was purchased from R&D Systems (Minneapolis, MN). Forskolin (10 mm in DMSO; S2449) was purchased from Selleck Chemicals (Houston, TX). Dialysis membranes (molecular mass cutoff = 3500 Da) (132724) were purchased from Spectrum Laboratories (Rancho Dominguez, CA). Endothelial growth media‐2 (EGM2; CC‐3162) was purchased from Lonza (Walkersville, MD) and supplemented with additional characterized HyClone FBS from GE Healthcare Life Sciences (HyClone FBS; SH30071.03) (Logan, UT) and used to culture ECFCs (provided by M. Yoder, Indiana University School of Medicine) on collagen I, rat tail (Col I; 354236) from Corning (Corning, NY) coated cell culture plates. SMARTpool:siGENOME MMP1 siRNA (M‐005951‐01‐005), siGENOME Non‐Targeting siRNA 1 (D‐001210‐01‐05), and DharmaFECT2 (T‐2002‐02) were purchased from Dharmacon Inc. (Lafayette, CO). Direct‐zol RNA Miniprep Kits (R2052) were purchased from Zymo Research (Irvine, CA).

### Synthesis of Gelatin‐g–Ferulic Acid

Gelatin‐g–ferulic acid was synthesized using EDC and NHS as coupling reagents. A mixture of DMSO and DI water (1:1 volume ratio) was prepared as a solvent. Gtn (1.0 g) was dissolved in 50 mL of the solvent at 40 °C. FA (0.777 g, 4.0 mmol) was dissolved in 20 mL of the solvent and reacted with EDC (0.92 g, 4.8 mmol) at room temperature for 15 min and then with NHS (0.64 g, 5.6 mmol) at room temperature for 15 min to activate the terminal carboxyl groups of FA (carboxyl/EDC/NHS = 1:1.2:1.4). The activated solution was then added to the Gtn solution, and a conjugative reaction was conducted at 40 °C for 24 h. Following completion of the reaction, the solution was dialyzed against DI water for 5 days (molecular mass cutoff = 3500 Da) and then lyophilized.

### Preparation of Hypoxia‐Inducible (O_2_‐Controllable) Hydrogels

Hydrogel precursor solutions (Gtn–FA, laccase, mTG) were prepared in DBPS. Enzymes were maintained at final concentrations of 25 U mL^−1^ (laccase) and 0.15–0.6 U mL^−1^ (mTG). Hypoxia‐inducible hydrogels were prepared by mixing aqueous Gtn–FA and laccase/mTG solutions. Hydrogels were prepared in 1.5 mL vials in a 3:1 polymer:enzyme ratio. Polymer solutions (Gtn–FA, 4.0 wt%) and enzyme solutions (100 U mL^−1^ laccase and/or 0.6–2.4 U mL^−1^ mTG) were mixed by pipetting to form hydrogels. All gels were formed at 37 °C.

### Computational Model of O_2_ Gradients

Oxygen gradients within O_2_‐controllable hydrogels were computed using a mathematical model based on models developed in the authors' previous reports.^[^ ^10^, ^32^ ^]^ In brief, the authors assumed reductions in oxygen were due to both the O_2_ consumption rate of the laccase‐mediated cross‐linking reaction and the cellular oxygen consumption rate, which both follow Michaelis–Menten kinetics. These reductions in oxygen were balanced by diffusion of atmospheric oxygen to yield an oxygen gradient. The authors simulated three‐layer models (cells in the bottom, middle, or top) over 24 h, and two‐layer models (cells in the bottom or top) of the oxygen gradients with commercially available software, Comsol Multiphysics. Optimal layer thickness was determined using a combinatorial approach, where O_2_ gradients were modeled using Comsol, and volumes which could be used to work around technical and biological limitations were investigated. If the volume (and thus gel layer thickness) was too low, the effects of the gel meniscus were severe, resulting in variable cell behavior and areas of the gel (in the center) that did not cover the entirety of the bottom of the well‐plate for bottom layers. If the volume was too high, variable cell behavior was observed. Optimum layer thickness resulted in layers with distinct levels of O_2_, as well as uniform cell behavior.

### O_2_ Measurements

Dissolved O_2_ (DO) levels were measured noninvasively in both acellular and cell‐encapsulated hydrogels at the bottom of hydrogels using commercially available sensor patches (Oxygen Sensor Spot; SP‐Pst3) and a multichannel fiber‐optic oxygen meter (OXY‐4 mini) from PreSens (Regensburg, Germany). O_2_ patch sensors were calibrated with manufacturer provided calibration values. To measure O_2_ levels at the bottom of hydrogels, the hydrogels were added on top of the sensors, which were immobilized in each well of a 96‐well plate. All experiments were conducted in a controlled environment at 37 °C and 5% CO_2_ in a standard incubator.

O_2_ gradients were measured in preformed cell‐encapsulated hydrogels at specified time points (continuous gradient measurements were not possible with the authors' O_2_ sensors). Commercially available needle‐type oxygen microsensors (Oxygen Microsensor; NTH‐Pst1) and a microfiber optic oxygen transmitter (Microx TX3) from PreSens were used to measure O_2_ gradients. Sensors were calibrated using atmospheric and anoxic (N_2_ flush) conditions at 37 °C. O_2_ sensors were precisely controlled using a Manual Micromanipulator MM (PreSens). Starting at the bottom of the hydrogel, measurements were recorded (when the reading stabilized) every 250 µm within the hydrogel and every 500 µm or 1 mm within the media.

### Cell Culture and Analysis in Hypoxia‐Inducible (O_2_‐Controllable) Hydrogels

All cells were cultured using standard, humidified cell culture incubators at 37 °C and 5% CO_2_, unless otherwise specified. ECFCs (Yoder Lab, Indiana University School of Medicine) were cultured in EGM2 (Lonza) prepared according to the manufacturer's instructions with an additional 10% HyClone FBS, on standard tissue culture plates coated with type I collagen (Corning).

### ECFC Encapsulation in Layered Hydrogels

ECFCs (Yoder Lab, Indiana University School of Medicine) were cultured in EGM2 (Lonza) prepared according to the manufacturer's instructions with an additional 10% HyClone FBS, on standard tissue culture plates coated with type I collagen (Corning). Cells were used between passages 7–10. For all cellularized hydrogel layers, polymer solutions were dissolved in 1× DPBS (pH 7.4) and mixed with ECFC pellets to provide a cell suspension (4 million cells/mL), and then enzyme solution (laccase—100 U mL^−1^; mTG—0.6–2.4 U mL^−1^) was added at a volume ratio of 3:1 (polymer solution/enzyme solution) and gently mixed at 37 °C for a predetermined “preincubation time.” Preincubation times for each batch of polymer were based on the gelation time, as measured by the vial‐tilt method. On the basis of this gelation time, a preincubation time was calculated (gelation time minus 1 min) to prevent all cells from falling to the bottom of the hydrogel, thus ensuring a homogeneous distribution of cells in all conditions.

Both two‐ and three‐layer hydrogels were generated. For both the two‐ and three‐layer hydrogels, 50 µL “bottom” layers containing cells (ECFCs or GFP‐ECFCs) were generated in the same manner as described above. Following the formation of this layer, hydrogels were incubated for 20 min at 37 °C. Then, a 50 µL (for the two‐layer system) or 100 µL (for the three‐layer system) layer of acellular hydrogel was added, then incubated for 20 min at 37 °C. Following incubation, 100–200 µL EGM‐2 was added. To generate the “top” layer, an acellular hydrogel (50 µL for the two‐layer system; 100 µL for the two‐layer system) was generated and incubated at 37 °C for 20 min, then a 50 µL cellularized layer was added atop the acellular hydrogel, and then incubated at 37 °C for 20 min. Following incubation, 100–200 µL EGM‐2 was added. To generate the “middle” layer in the three‐layer hydrogels, a 50 µL acellular hydrogel was generated and incubated at 37 °C for 20 min, then a 50 µL cellularized layer was added atop the acellular hydrogel, then incubated at 37 °C for 20 min, then a 50 µL acellular layer was added atop the hydrogel, and then incubated at 37 °C for 20 min. Following incubation, 100–200 µL EGM‐2 was added. Cells were cultured under standard cell culture conditions (37 °C, 5% CO_2_). The culture medium was replaced daily. Bright‐field images were captured at predetermined time points to monitor cell morphology using an Olympus IX50 (Olympus; Center Valley, PA).

### RNA Extraction and Purification

RNA was extracted from ECFCs cultured in “bottom” (hypoxic) layers or nonhypoxic conditions. EGM‐2 was removed and 200 µL Trizol reagent was added atop the hydrogels and incubated at room temperature for 5 min. Hydrogels were then mixed using a P1000 pipette tip and transferred to a 1.5 mL microfuge tube containing an additional 300 µL Trizol reagent. Samples were then homogenized using a pellet pestle homogenizer (FisherBrand) for 1–2 min. Samples were then centrifuged at 12,000 × *g* for 10 min at 4 C. The supernatant was then transferred to a fresh 1.5 mL microfuge tube and stored at −80 °C until ready for purification. Samples were thawed on ice prior to purification. RNA was purified using Direct‐zol RNA Miniprep kits according to the manufacturer's suggestion (Zymo Research; Irvine, CA), with a few minor exceptions and clarifications identified in the following text. All centrifugation steps were performed at 13,000 × *g* for 30 s at 4 °C, unless another speed was specified. DNase I treatment was used. Samples were eluted in 15 µL DNase/RNase‐free water. Following elution, the samples were reloaded and eluted a second time. Following purification, samples were stored at −80 °C.

### RNA‐Sequencing

RNA sequencing was performed at the Johns Hopkins University School of Medicine Transcriptomics and Deep Sequencing Core, guided by the direction of Dr. Haiping Hao. RNA‐seq library for Illumina platform sequencing was prepared using Illumina TruSeq stranded total RNA Sample kit following manufacturer's recommended procedure. Briefly, 100 ng of total RNA was first depleted of ribosomal RNA with Ribozero Gold magnetic beads and further purified with Agencourt AMPure XP beads. RNA was fragmented at 94 °C for 8 min and primed with random primer. The fragmented RNA was then converted to double strand cDNA, end repaired, A tailed, and ligated with Unique Dual Indexed adaptors. The adaptor added cDNA library was then PCR amplified using the following conditions: 94 °C for 30 s, 15 cycles of 98 °C for 10 s, 60 °C for 30 s, 72 °C for 30 s, and 72 °C for 5 min. The PCR amplified library was purified using Agencourt AMPure XP magnetic beads and run out on Agilent High Sensitivity DNA Chip for quality check. Individual library was then further quantified using KAPA library quantification qPCR kit and pooled. The pooled library was sequenced on Illumina NovaSeq S1 200cycle kit for 2X100bp sequencing.

### Analysis of RNA‐Sequencing Data

RNA sequencing analysis was performed at the Johns Hopkins University School of Medicine Transcriptomics and Deep Sequencing Core, by Conover Talbot. RNA 100 base paired‐end sequencing libraries were constructed from 24 samples of total RNA using the Illumina TruSeq Stranded mRNA kit following manufacturer's protocol. Following QC, the libraries were sequenced on the Illumina NovaSeq 6000 System using their S1 flow cell and base calling performed with RTA version 2.4.11. Sequencing adapters were stripped with the bcl2fastq 2.17.1.14 program, which provided 48 raw FASTQ files representing the 24 biological samples. Using the CLC Genomics Server 9.1.1 these reads were aligned to the NCBI April 2019 transcriptome, GRCh38.p13, of 54136 transcripts. The transcript identifiers were derived from these FPKM files’ “Name” column and were updated to current HGNC/NCBI nomenclature.

The raw data files’ FPKM values of 0.0 were treated as nulls and the remaining actual values transformed into log2 notation. QC examination of the raw log2 signals in box plot and histogram showed technically consistent sequencing results across all 24 samples. PCA figures showed clean separation of the biological classes. Two samples, however, clustered unequivocally out of their expected classes and were therefore excluded from further analysis.

The remaining 22 samples’ log2 signal values were quantile normalized together for further analysis. The six biological classes’, three time points with and without hypoxia, underwent differential expression analysis with a two‐tailed one way *t*‐test ANOVA using the Partek GS 7.0 7.18.0723 platform.

The results report transcripts’ differential expression in ratio, linear fold change, and log2 fold change notation, and their statistical significance in uncorrected *p*‐values. The original FPKM files’ “XLOC_###” and gene annotations were retained as well as updated NCBI/HGNC gene nomenclature annotation.

A standard deviation analysis was performed for each class–class comparison using only those transcripts that had: NCBI Entrez gene anotation, a mean FPKM log2 value >−0.8 in the higher‐expressed assayed biological class. On the order of 11K transcripts met these criteria for each cell–class comparison. The accession number for the gene expression profiles described here is GSE17612.

### CellROX for Oxidative Stress Detection

CellROX was used to identify oxidative stress in live cells. CellROX stock solutions were prepared at 2.5 mm in DMSO. CellROX (5 µm) was then co‐encapsulated with ECFCs in layered hydrogels and fluorescence intensity was monitored over 24 h at predetermined time points using an Olympus IX50 (Olympus; Center Valley, PA) for brightfield and fluorescent images.

### Forskolin for Inhibition of Cluster Formation

Forskolin stock solution at 10 mm in DMSO was used to inhibit cluster formation in hydrogels. Confluent flasks of ECFCs were incubated in 10 mL EGM‐2 with 500 µm forskolin at 37 °C for 1 h. Following the incubation, cells were co‐encapsulated with 500 µm forskolin in hypoxic and nonhypoxic hydrogels. After incubating at 37 °C for 20 min, 100–200 µL EGM‐2 with 500 µm forskolin were added atop the hydrogels. Bright‐field images were captured at predetermined time points to monitor cell morphology using an Olympus IX50 (Olympus; Center Valley, PA).

### siRNA Transfection

ECFCs were transfected with SMARTpool:siGENOME MMP1 and Non‐Targeting siRNA 1 (scr) according to the manufacturer's protocol and previous literature.^[^ ^10^ ^]^ Briefly, cells were seeded on a six‐well plate and treated with 50 nm siRNA. The authors confirmed knockdown via quantitative real‐time fluorescence polymerase chain reaction after 24 h and used transfected cells in experiments.

### Protease Inhibition (Z‐FF‐FMK, PI, EDTA)

Stock solutions were prepared according to manufacturer's instructions in the appropriate solvent. Hydrogel precursor solutions and/or ECFC medium (EGM2) were supplemented with Z‐FF‐FMK (final concentration 10–500 µm), ProtInh (final concentration 0.1×), EDTA (final concentration 0.1×), or ProtInh/EDTA (final concentration 0.1×), and bright‐field images were captured at predetermined time points to monitor cell morphology and cluster formation or inhibition (Olympus IX50).

### Immunostaining

After 24 h in culture, ECFCs within Gel‐HI hydrogels were fixed with 2% FA for 20 min at room temperature. Hydrogels were then washed thrice with 1× DPBS with 10 min in between each wash. Encapsulated ECFCs were then permeabilized with 1% Triton X‐100 for 15–20 min and then washed thrice with 1× DPBS with 10 min in between each wash. Next, hydrogels were incubated in 5% BSA blocking solution for 1 h at room temperature and then washed with 0.05% Tween‐20 in 1× DPBS thrice with 10 min in between. Hydrogels were then stained with primary antibody in antibody diluent solution overnight at 4 °C and then washed with 0.05% Tween‐20 in 1× DPBS thrice with 10 min in between. Hydrogels were incubated in secondary antibody and phalloidin in antibody diluent solution for 2 h at room temperature and then washed with 0.05% Tween‐20 in 1× DPBS thrice with 10 min in between. Last, hydrogels were incubated in DAPI solution for 15 min at room temperature and then washed with 1× DPBS thrice with 10 min in between. Hydrogels were analyzed using confocal microscopy (LSM 780; Zeiss). Primary antibody VCAM‐1 (R&D Systems) was diluted in sterile DBPS and was used at a final concentration of 12 µg mL^−1^ in antibody diluent (Thermo Fisher Scientific), according to the manufacturer's protocol. Secondary antibodies, ms488, were used at 1:250 in antibody diluent, and phalloidin was used at 1:500 in antibody diluent.

### Cell Cycle Flow Cytometry

Col. IV solution, prepared at 5 mg mL^−1^ in DPBS, was used to dissolve cellularized hydrogels. BSA was dissolved in DPBS to 0.1% v/v to prepare BSA in DPBS. PI staining solution was prepared by diluting the PI reagent to 2 µg mL^−1^ in DPBS with BSA. 100 µg mL^−1^ RNase A was added to reduce background stain.

At predetermined time points, EGM‐2 was removed and 100–200 µL DPBS was added atop the hydrogels. After removing the DPBS, 100–200 µl of Col. IV solution was added, then incubated at 37 °C for 12 min. Cells from three wells were combined into a single 1.5 mL microfuge tube and spun down at 200 × *g* for 5 min. After removing the supernatant by aspirating, cell pellet was resuspended in 1 mL DPBS, gently vortexed, then spun down at 200 × *g* for 5 min. Supernatant was removed by aspirating, and cell pellet was resuspended in 300 µL DPBS. Finally, 700 µL ice‐cold sterile 100% EtOH was slowly added to the cells. Cells were vortexed gently and stored at 4 °C overnight.

The fixed cells were spun down at 400 × *g* for 5 min. Supernatant was removed by aspirating, and pellet was resuspended in 1 mL DPBS. Cells were gently vortexed and spun down at 800 × *g* for 5 min. Supernatant was removed by aspirating, and pellet was resuspended in 1 mL DPBS. Cells were gently vortexed and spun down at 800 × *g* for 5 min. After aspirating the supernatant, fixed cell pellet was resuspended in 500 µL PI staining solution. Tubes were wrapped in foil and incubated at room temperature for 10 min. Cells were filtered through a strainer cap into a 12 × 75 FACS tube.

The stained cells were measured on Becton Dickinson FACSCanto (Becton Dickinson; Franklin Lake, NJ) for cell cycle at the Johns Hopkins University Integrated Imaging Center. The data were analyzed through FCS Express 7 (De Novo Software), where the measurements were fitted to a statistical model SL S0 to account for the debris. As the debris curve resembled a ski slope and as the S order resembled a broadened rectangle, SL S0 was selected for analysis. *T*‐test was performed on GraphPad Prism 8 (GraphPad Prism Software Inc.).

### Statistical Analysis

The authors performed statistical analysis using GraphPad Prism 8 (GraphPad Software Inc.). This software was also used to perform *t*‐tests and one‐way ANOVA to determine significance. Replicates were indicated throughout the figure captions. All graphical data were reported as means ± SD. Significance levels were set at *p < 0.05, **p < 0.01, ***p < 0.001, and ****p < 0.0001. All graphical data were reported. Statistical analysis for sequencing data is described above.

## Conflict of Interest

The authors declare no conflict of interest.

## Supporting information

Supporting Information

## Data Availability

The data that support the findings of this study are available from the corresponding author upon reasonable request.
