# Supplementary material for: Discretizing Three‐Dimensional Oxygen Gradients to Modulate and Investigate Cellular Processes
Source: Adv Sci (Weinh). 2021 Jun 21;8(14):2100190. doi: 10.1002/advs.202100190 (PMC8292886; doi:10.1002/advs.202100190)
Supplement: Supplementary file 1 — Supporting Information [file ADVS-8-2100190-s001.pdf]

© 2021 Wiley-VCH GmbH

ADVANCED SCIENCE
Open Access

Supporting Information

for *Adv. Sci.,* DOI: 10.1002/advs.202100190

Discretizing 3D Oxygen Gradients to Modulate and Investigate Cellular Processes

*Michael R. Blatchley, Franklyn Hall, Dimitris Ntekoumes, Hyunwoo Cho, Vidur Kailash, Rafael Vazquez-Duhalt, Sharon Gerecht**

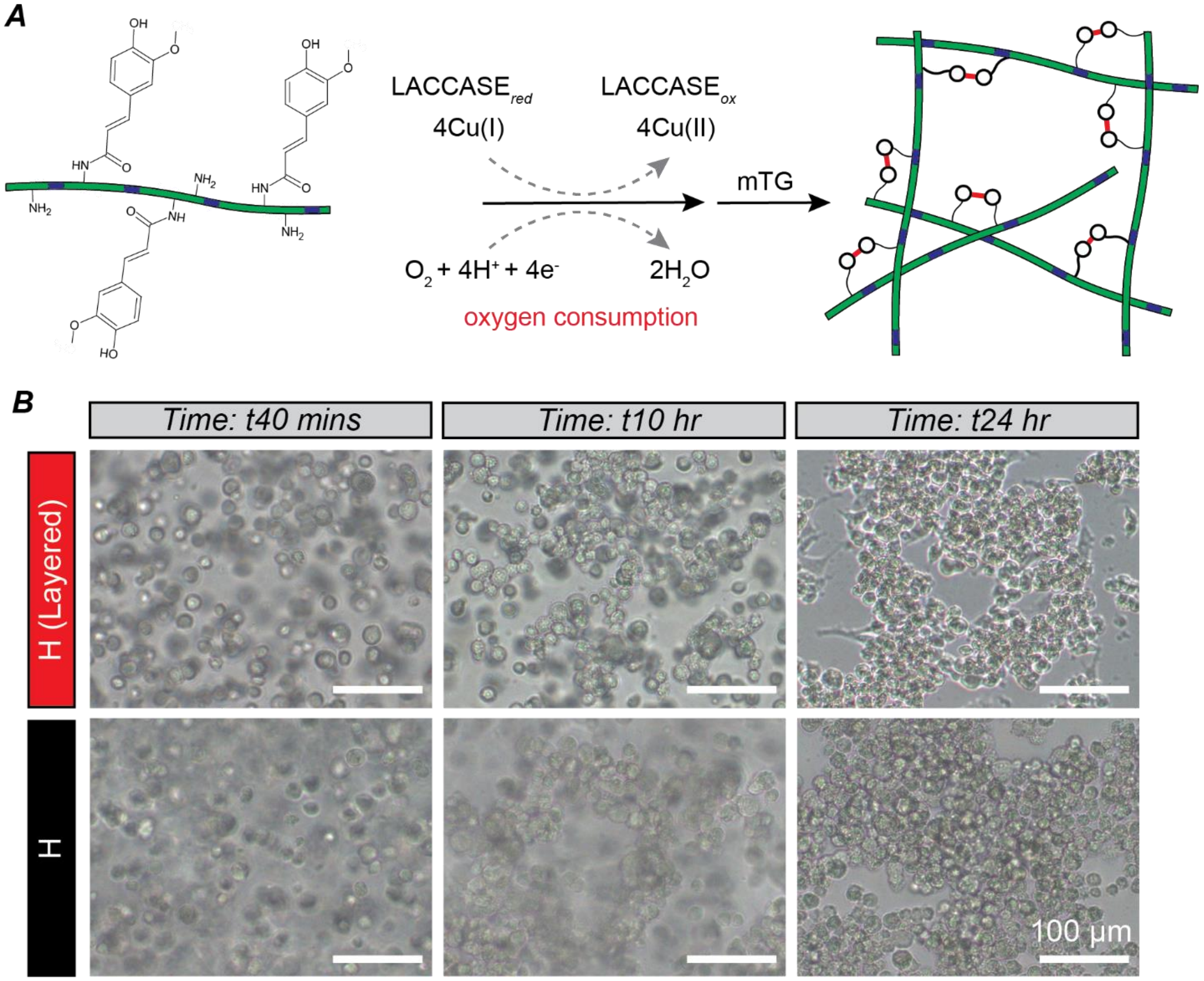

Figure S1.O_2-controllable hydrogel design and layered gel implementation. (A) Gelatin-based O_2-controllable hydrogels are formulated by conjugation of phenol-containing ferulic acid to a gelatin backbone, which is enzymatically crosslinked by O_2-consuming laccase and microbial transglutaminase (mTG). (B) Cluster formation kinetics were matched in the two hydrogel platforms, with clusters forming at 10 hr after encapsulation in both conditions, and clusters growing in terms of number of cells in clusters by 24 hr. Scale bars are 100 µm.

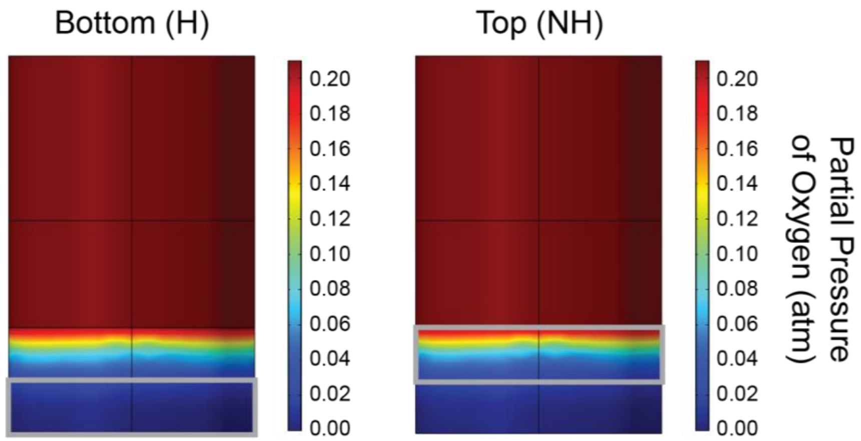

Figure S2. Computational modeling of 2-layer O_2-controllable hydrogels. Modeling of partial pressure of oxygen in the cell-laden bottom (hypoxic; H) and top (nonhypoxic; NH) layers of O_2-controllable hydrogels. Cell layers identified by gray boxes.

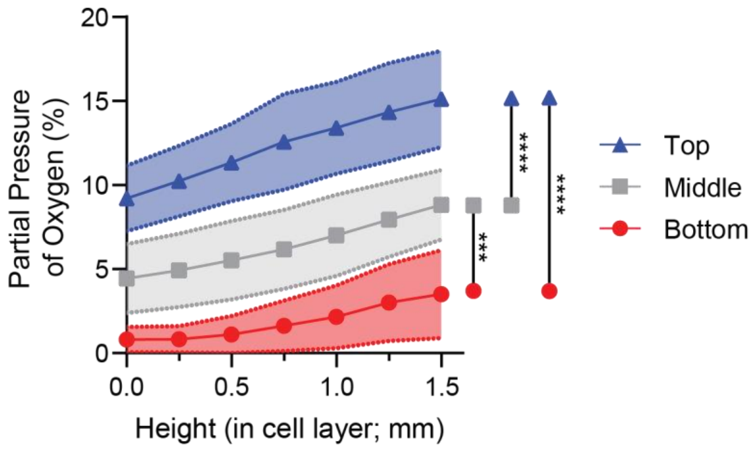

Figure S3. 3-layer hydrogel O_2 gradients. Gradient measurements were taken across the cellularized layer in bottom, middle, and top layers of 3-layer hydrogels. N=5 hydrogels per condition. ***p<0.001, ****p<0.0001.

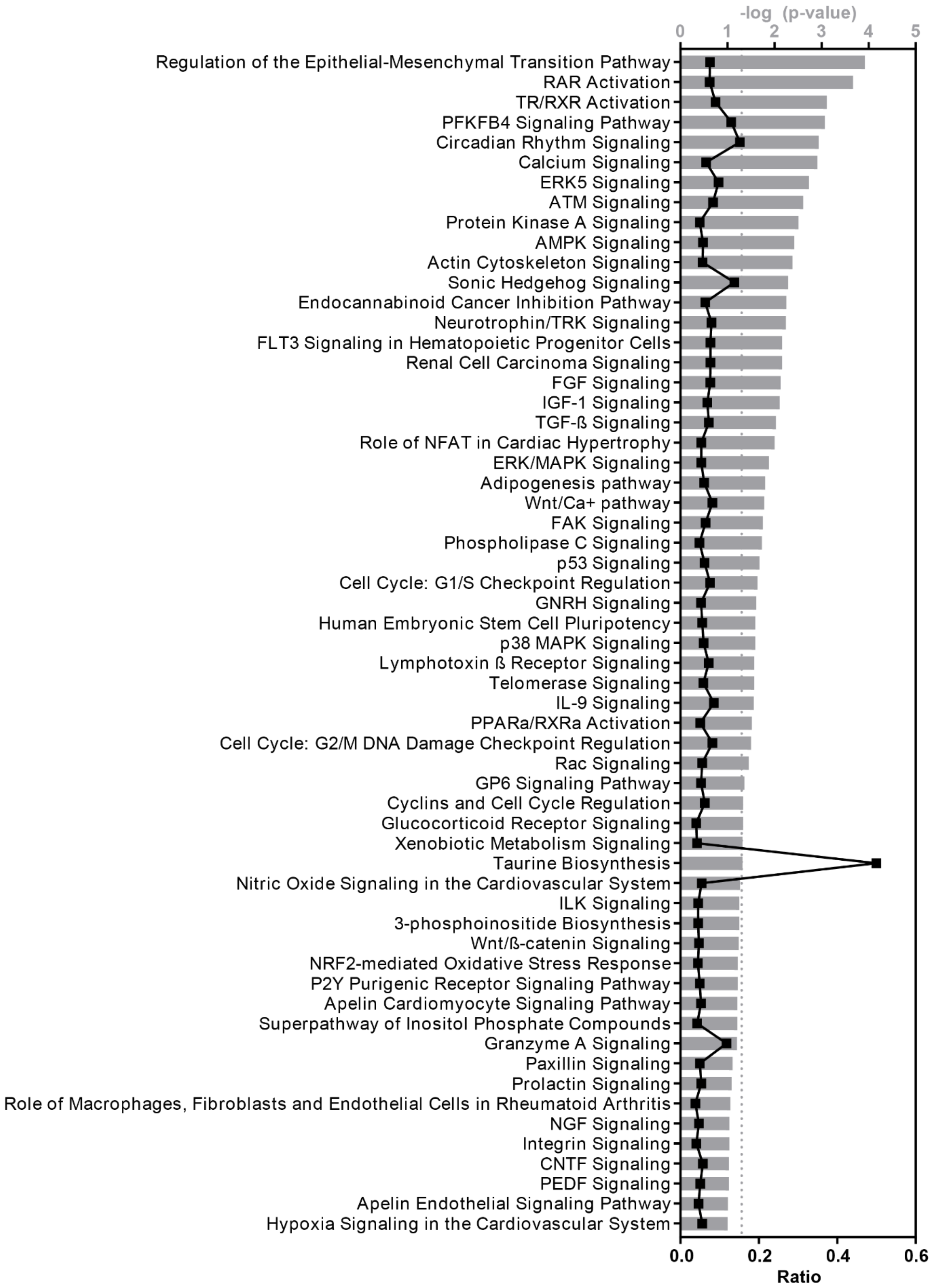

Figure S4: 40 mins time point IPA Canonical Pathway top hits. See Supplementary Data 2 and Experimental Section for additional information. IPA pathways at the 40 min time point. All pathways with a value greater than the dotted gray line have statistically significant overlap. Those with a below the gray line were curated by relevancy to our work. Ratios indicate the number of genes in our dataset compared to the total number of genes in each pathway.

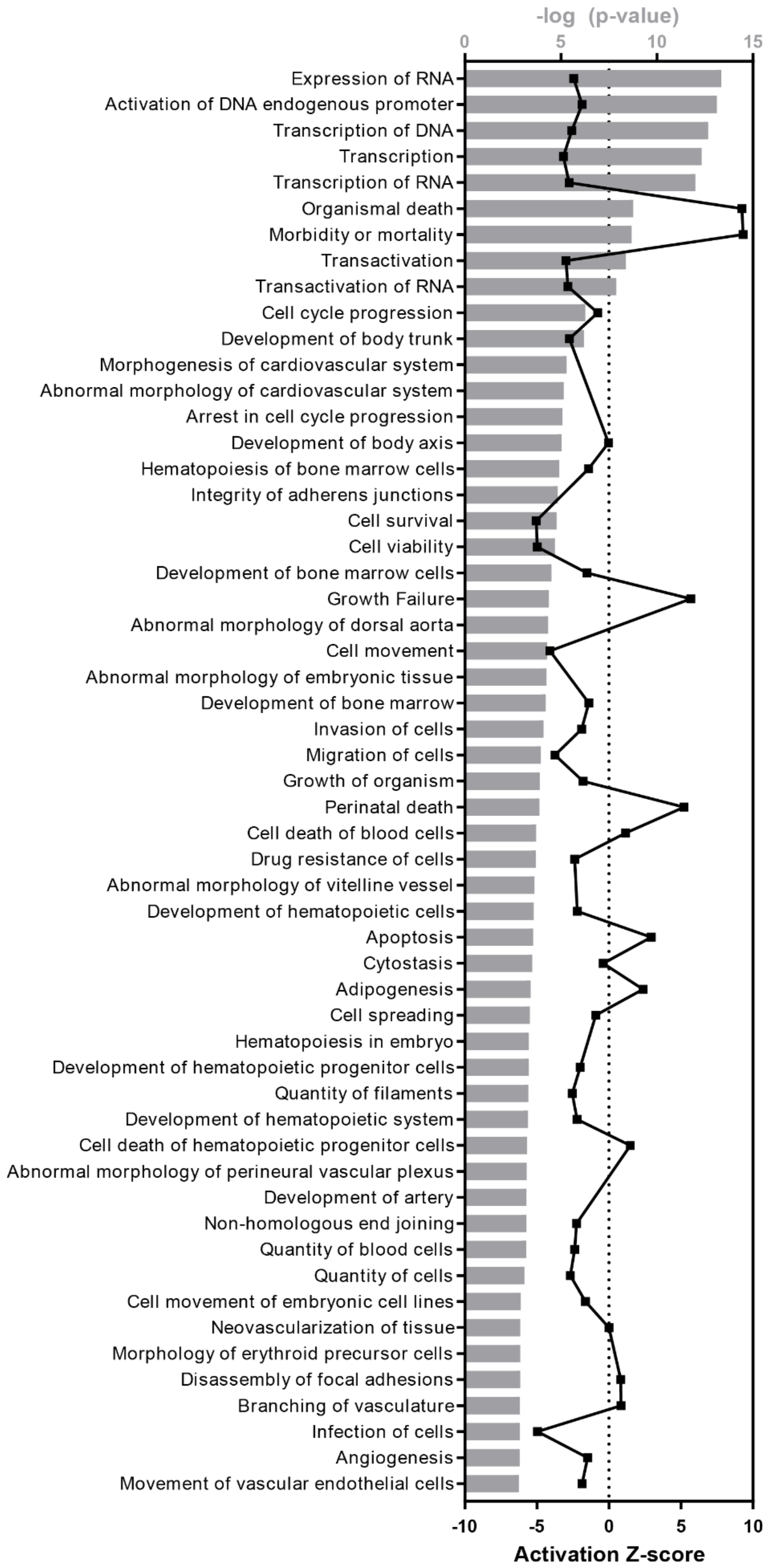

Figure S5: 40 mins time point IPA Biological Function top hits. See Supplementary Data 2 and Experimental Section for additional information. IPA functions at the 40 min time point. All functions presented are statistically significant. Activation Z-score indicates activation (+ value) or deactivation (- value). Functions without a Z-score black square did not have a calculated Z-score.

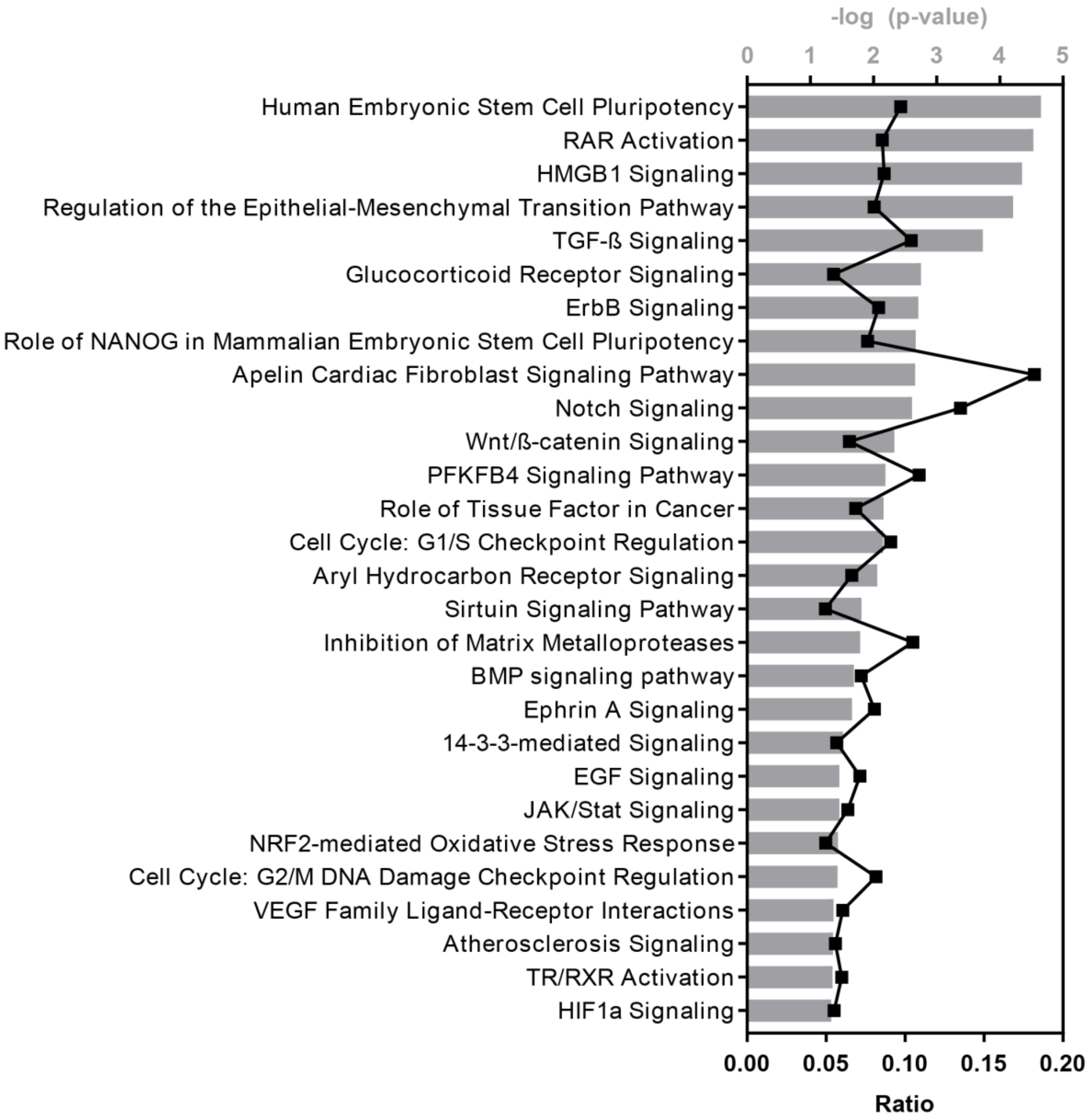

Figure S6: 10 hrs time point IPA Canonical Pathway top hits. See Supplementary Data 3 and Experimental Section for additional information. IPA pathways at the 10 hrs time point. All pathways have a statistically significant overlap. Ratios indicate the number of genes in our dataset compared to the total number of genes in each pathway.

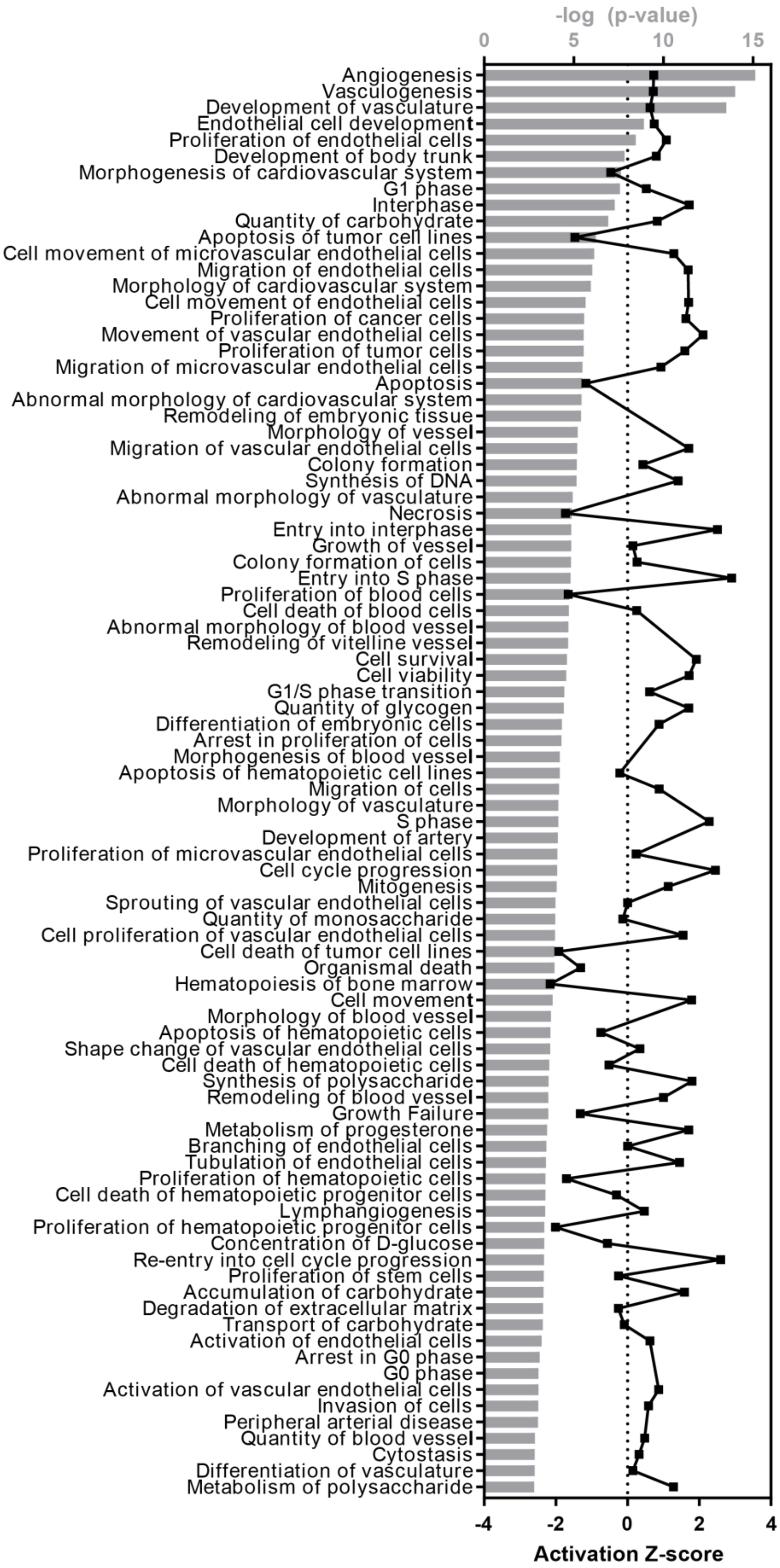

Figure S7: 10 hrs time point IPA Biological Function top hits. See Supplementary Data 3 and Experimental Section for additional information. IPA functions at the 10 hrs time point. All functions presented are statistically significant. Activation Z-score indicates activation (+ value) or deactivation (- value). Functions without a Z-score black square did not have a calculated Z-score.

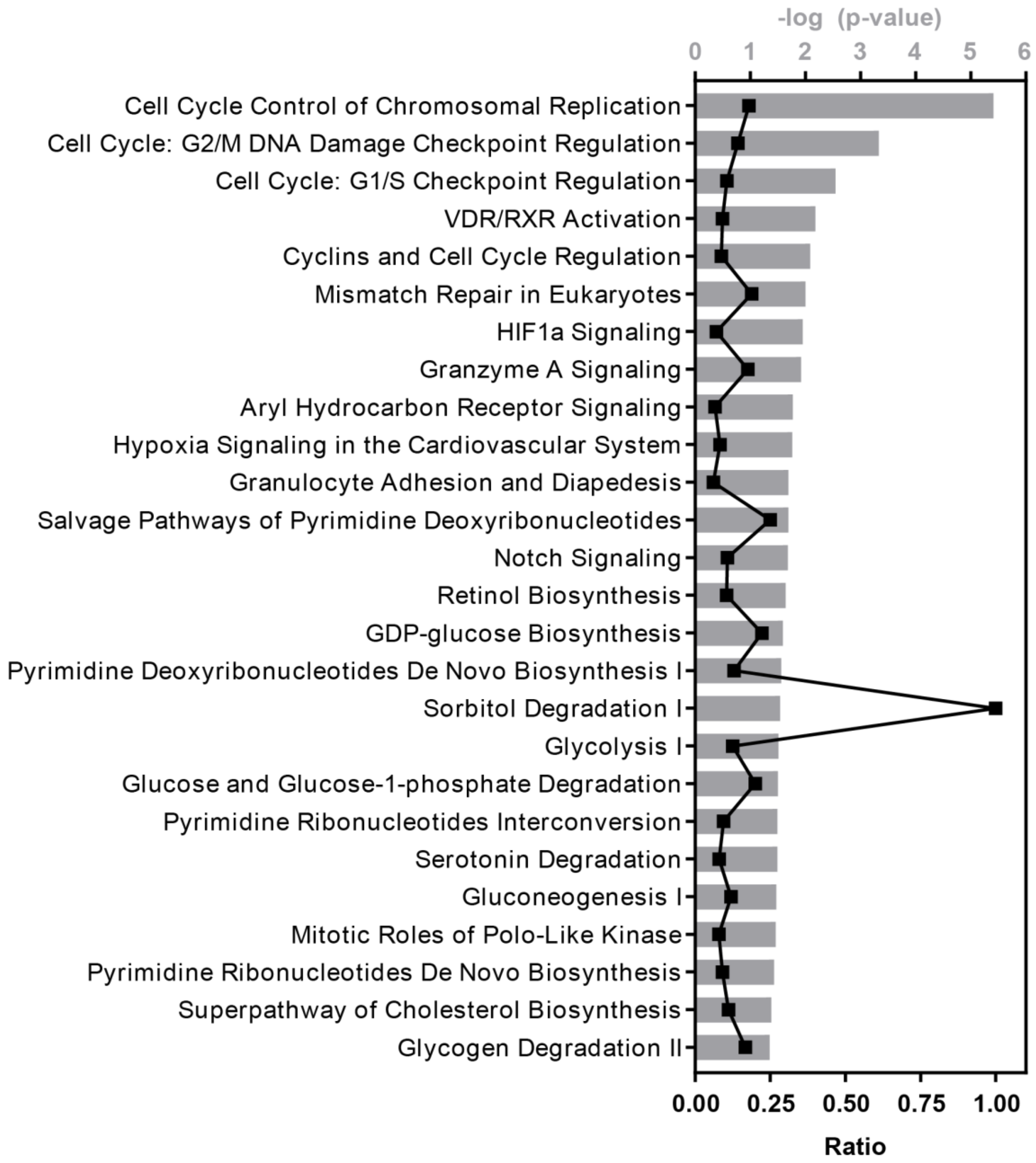

Figure S8: 24 hrs time point IPA Canonical Pathway top hits. See Supplementary Data 4 and Experimental Section for additional information. IPA pathways at the 24 hrs time point. All pathways have a statistically significant overlap. Ratios indicate the number of genes in our dataset compared to the total number of genes in each pathway.

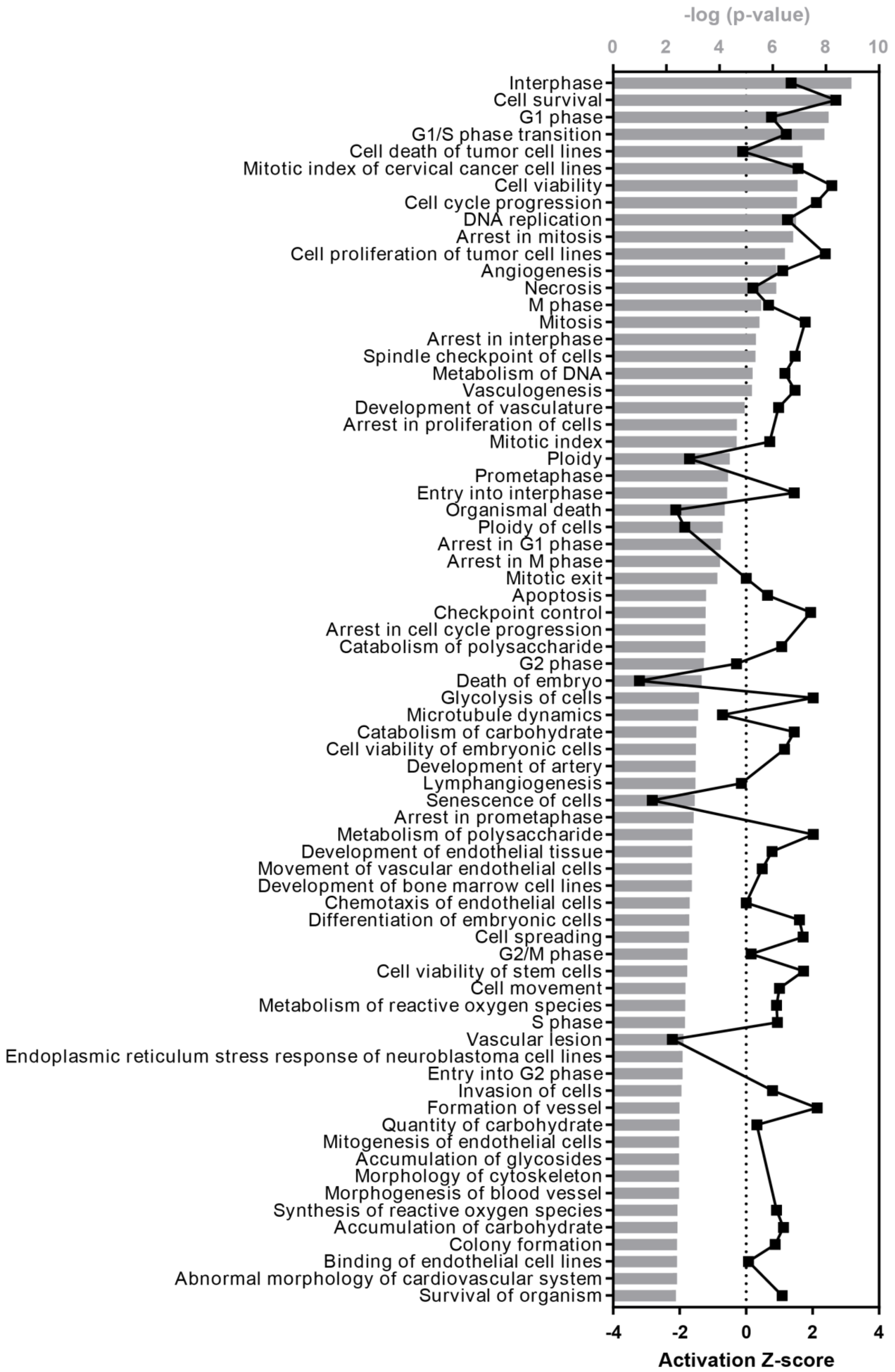

Figure S9. **24 hrs time point IPA Biological Function top hits**. See Supplementary Data 4 and Experimental Section for additional information. IPA functions at the 24 hrs time point. All functions presented are statistically significant. Activation Z-score indicates activation (+ value) or deactivation (- value). Functions without a Z-score black square did not have a calculated Z-score.

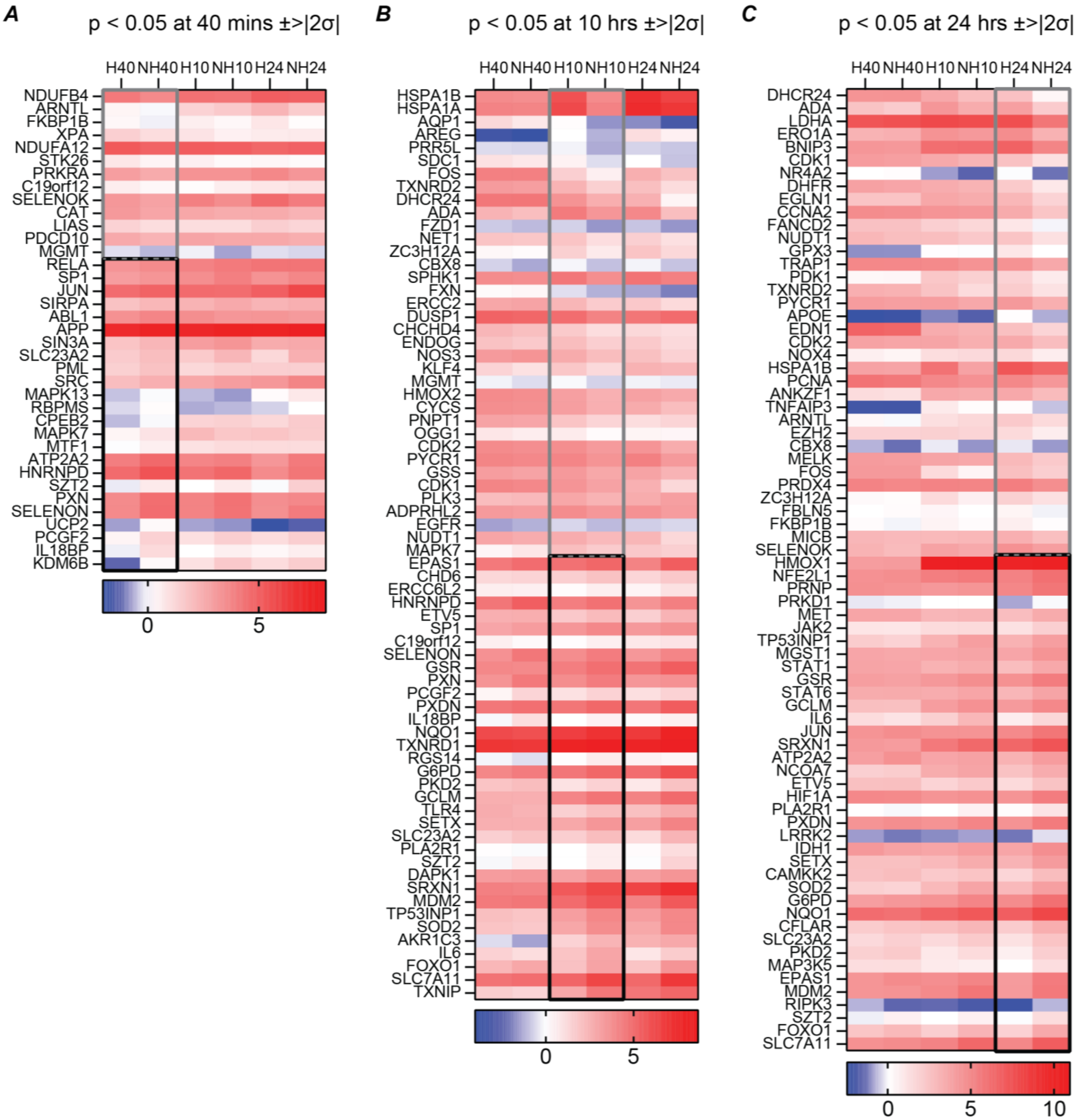

Figure S10. GO: 0006979 (response to oxidative stress) differentially expressed genes over the experimental time course. (A-C) Mean $\log_2$(FPKM) at each time point for all genes with p-value < 0.05 and standard deviation analysis value >l2σl. Gray boxed areas have positive $\log_2$(FC) and black boxed areas have negative $\log_2$(FC).

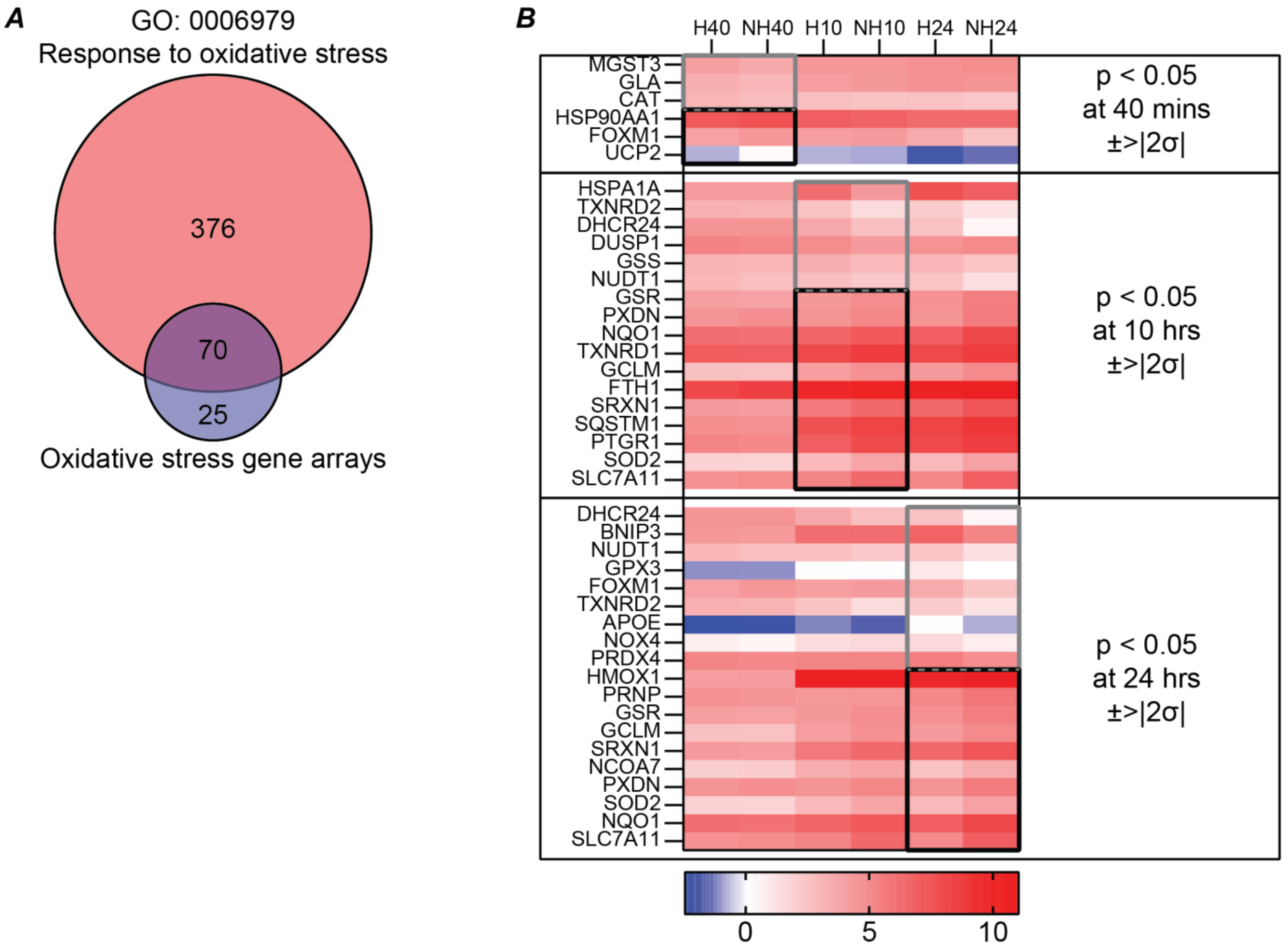

Figure S11. Oxidative stress gene array analysis. (A) Overlap between GO: 0006979 and commercially available oxidative stress gene arrays (https://geneglobe.qiagen.com/product-groups/rt2-profiler-pcr-arrays). (B) Mean $\log_2$(FPKM) at each time point for all genes with p-value < 0.05 and standard deviation analysis value >l2σl. Gray boxed areas have positive $\log_2$(FC) and black boxed areas have negative $\log_2$(FC).

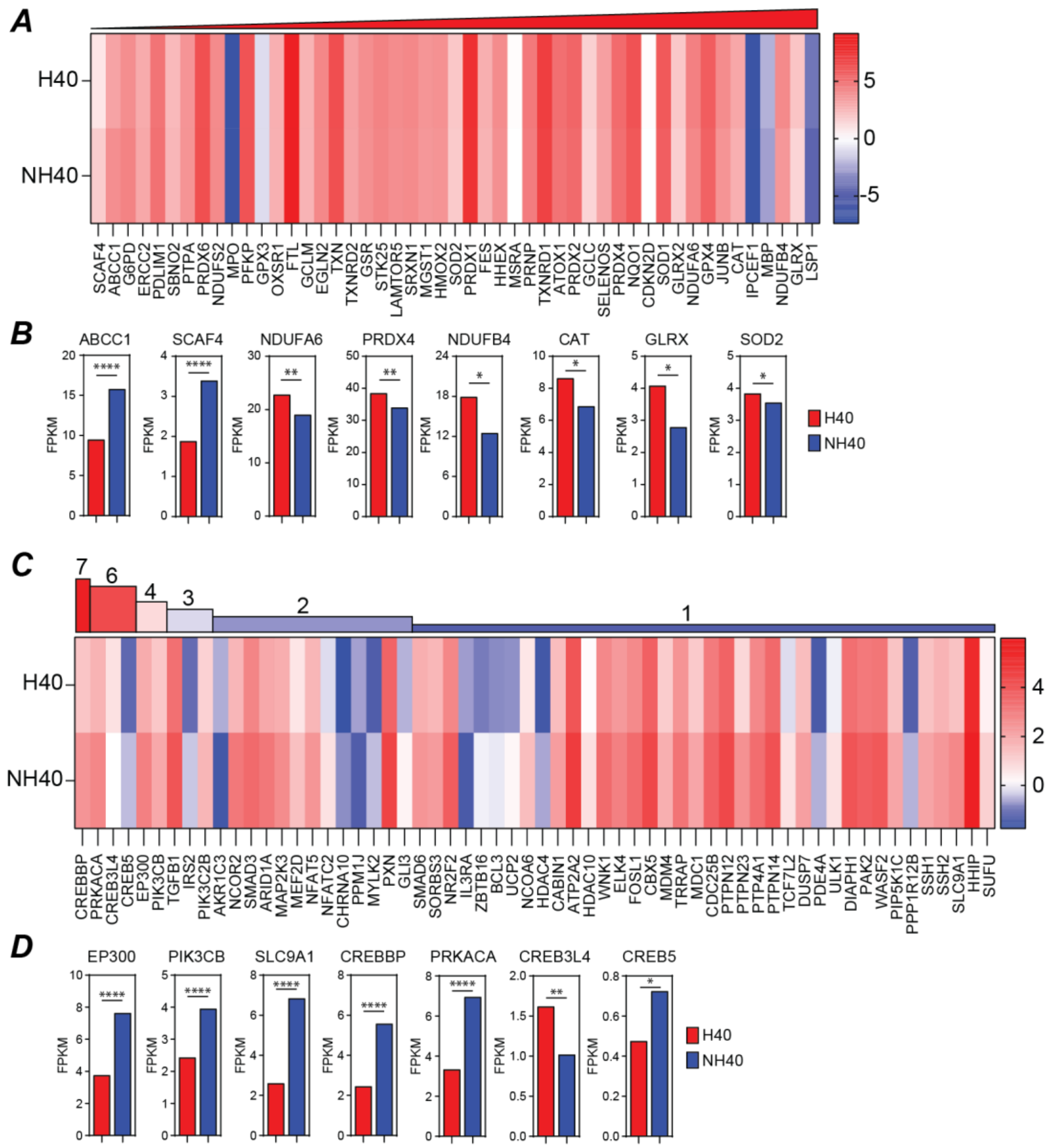

Figure S12. GSEA Hallmark reactive oxygen species genes and top IPA genes at the 40 mins time point. (A) $\log_2$(FPKM) of all genes in the Hallmark Reactive Oxygen Species Pathway. Left to right denotes increasing $\log_2$(FC). (B) Top differentially expressed genes based on p-value. (C) $\log_2$(FPKM) of genes identified by IPA. Ordered from left to right by number of pathways (in Figure 4C) with which each gene is associated. (D) Top differentially expressed genes from (C), as relevant to cAMP signaling. *$p<0.05$, **$p<0.01$, ***$p<0.001$, ****$p<0.0001$.

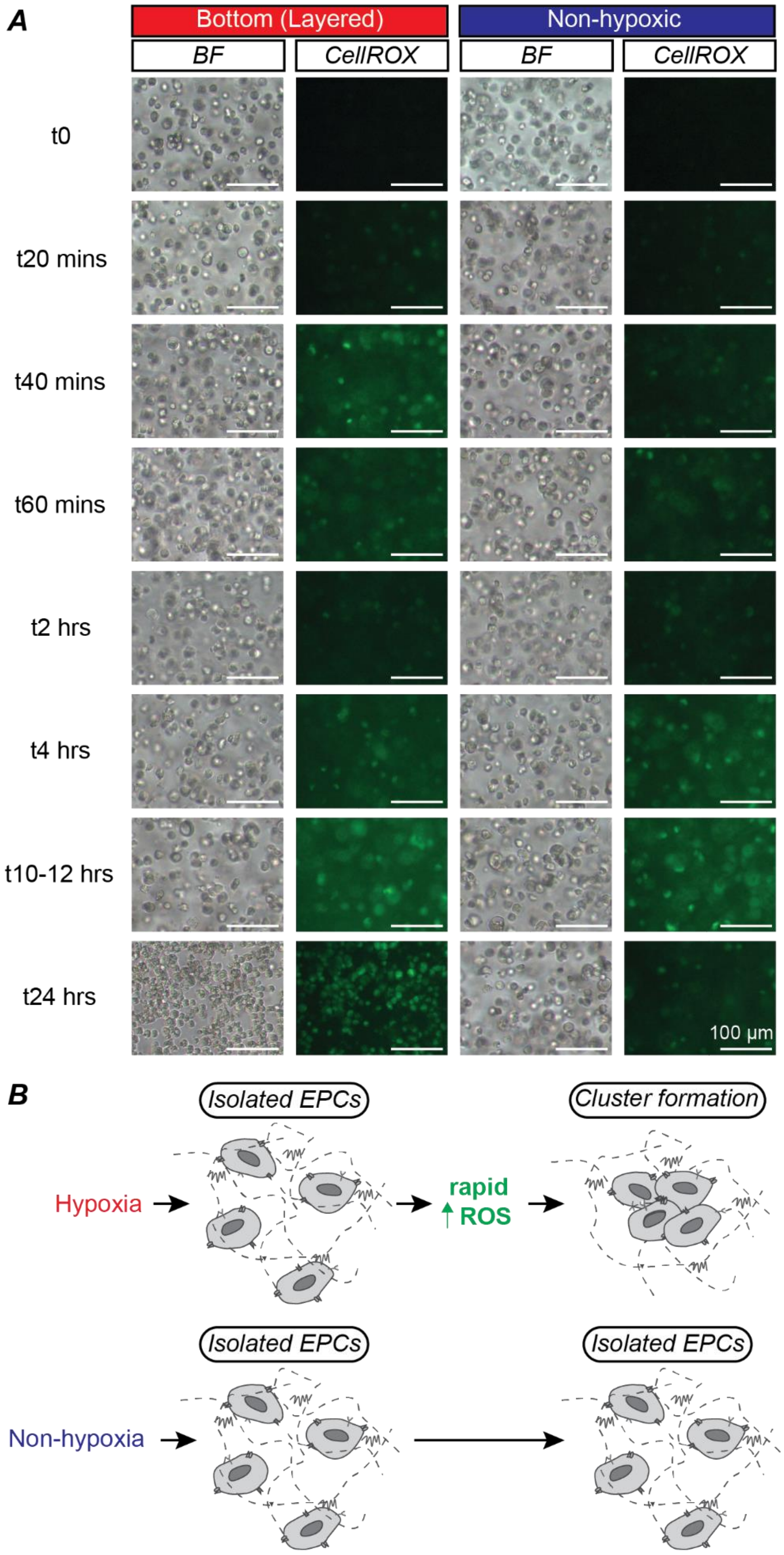

Figure S13. Oxidative stress is upregulated at early timepoints in ECFCs cultured in layered hypoxic hydrogels compared to nonhypoxic hydrogels. (A) ECFCs under both conditions were CellROX+ over the time course of the experiment, but CellROX increased at early timepoints under hypoxic conditions.(B) Rapid exposure to hypoxia leads to rapid ROS production, resulting in cluster formation in hypoxic conditions.

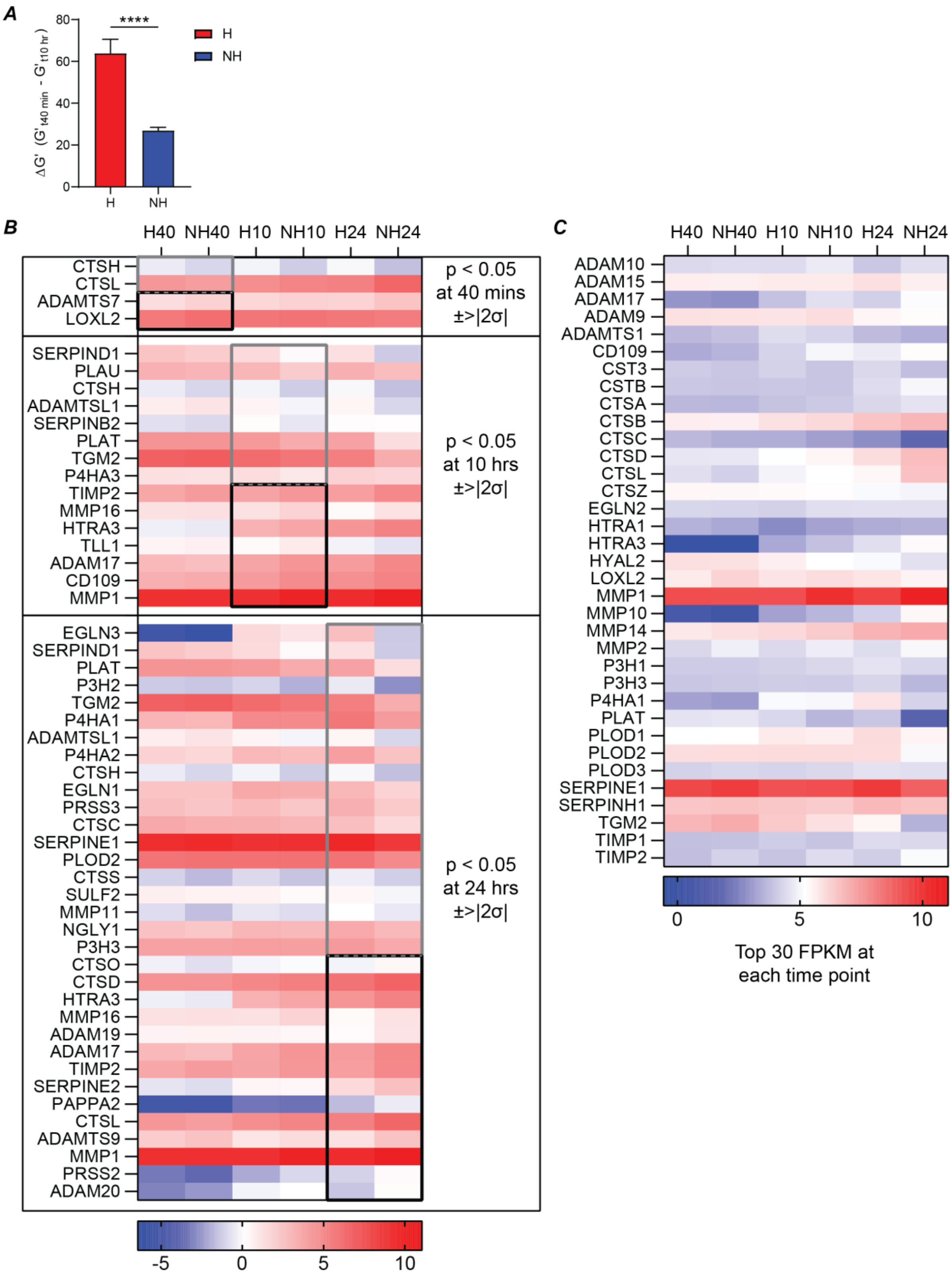

Figure S14. Matrix degradation upon cluster formation and differential expression of matrisome-associated regulators. (A) Rheological characterization of hypoxic (H) and non-hypoxic (NH) hydrogels. Data are presented as a change in G' from the 40 minute time point to the 10 hour time point. Both conditions result in a reduction in G' over time, with H conditions resulting a significantly increased reduction in G' over 10 hours. N=3 ($H_{t40\ mins}$), N=6 ($H_{t10\ hrs}$), N=4 ($NH_{t40\ mins}$), N=4 ($NH_{t10\ hrs}$). ****P<0.0001. (B) Mean $\log_2$(FPKM) at each time point for all genes with p-value < 0.05 and standard deviation analysis value >|2σ|. Gray boxed areas have positive $\log_2$(FC) and black boxed areas have negative $\log_2$(FC). (C) Mean $\log_2$(FPKM) at each time point for the top 30 FPKM (regardless of differential significance) at each time point. These genes were not necessarily differentially expressed, but had high read counts and thus may contribute to matrix remodeling.

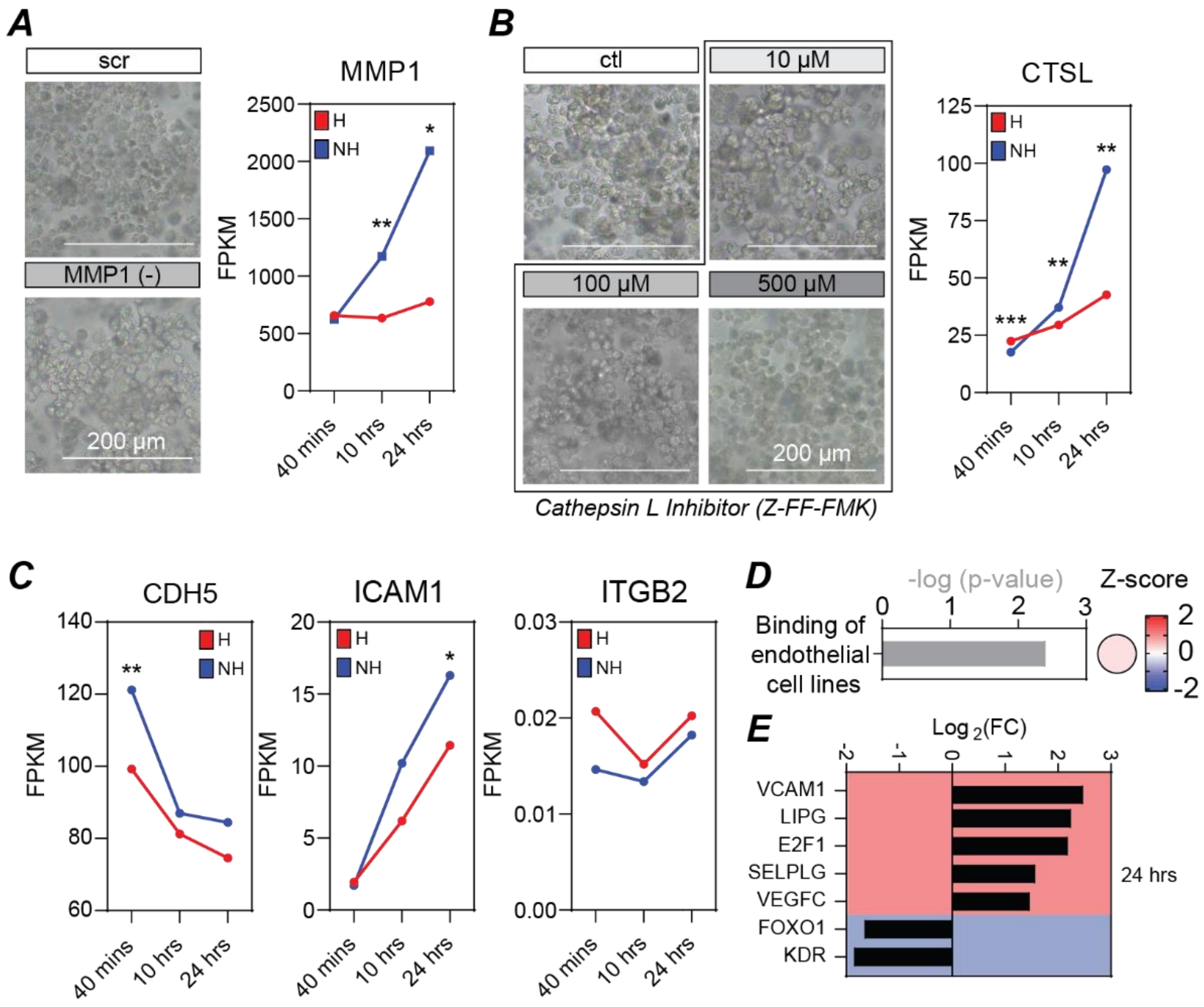

Figure S15.Proteases and cell-cell interactions are differentially regulated over the experimental time course. (A) siRNA knockdown of MMP1 did not inhibit cluster formation. FPKM of MMP1 over the experimental time course. (B) A range of concentrations of CTSL inhibitor Z-FF-FMK did not inhibit cluster formation. FPKM of CTSL over the experimental time course. (C) CDH5, ICAM1, ITGB2 FPKM over the experimental time course. (D) IPA biological function “binding of endothelial cell lines” at the 24 hr time point. P-value indicates statistically significant overlap; positive Z-activation score indicates activation. (E) Differentially expressed genes in the binding of endothelial cell lines IPA analysis. All genes are statistically significantly differently expressed and have a value of >l2σl based on standard deviation analysis at the 24 hr time point.*p<0.05, **p<0.01, ***p<0.001.

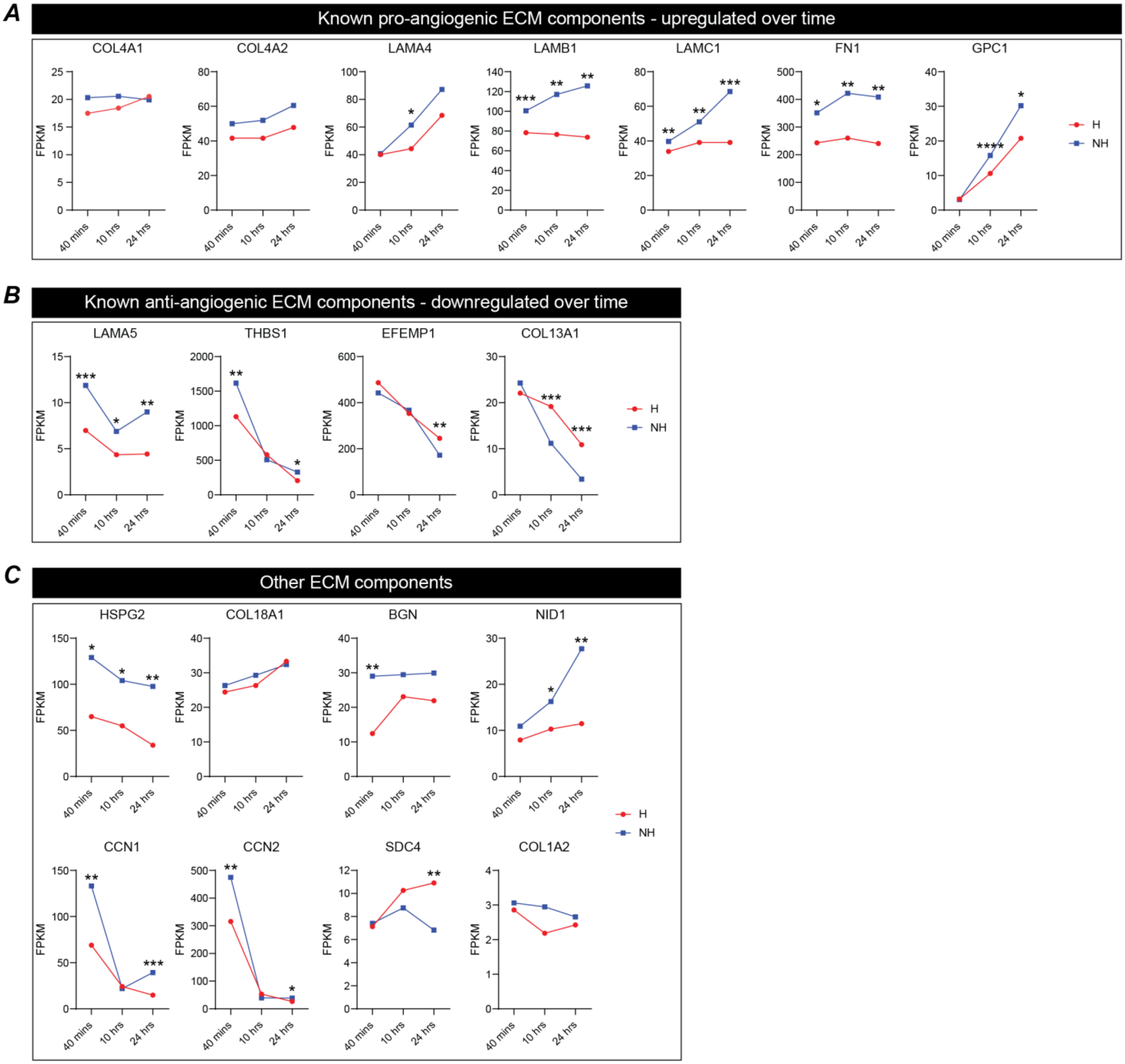

Figure S16. ECM characterization over time. (A) Genes encoding for pro-angiogenic ECM components upregulated over time. (B) Genes encoding for anti-angiogenic ECM components downregulated over time. (C) Genes encoding for other ECM components are differentially regulated over time. *P<0.05, **P<0.01, ***P<0.001, ****P<0.0001.

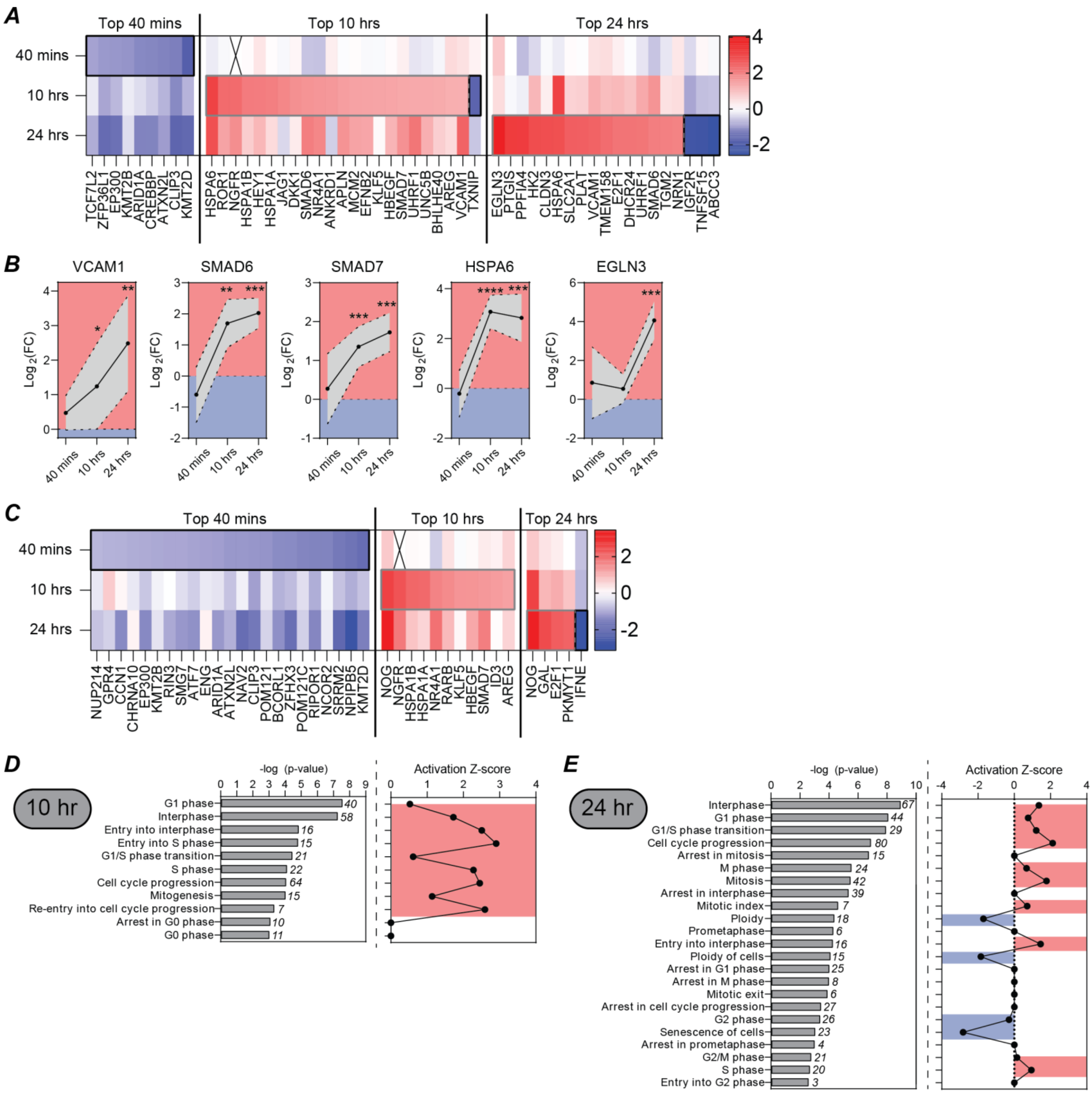

Figure S17. Differential expression of genes associated with cell survival and cell cycle contribute to cluster formation. (A)Significantly differentially expressed genes associated with cell survival over the time course of the experiment (≥l6σl at one or more time point, by standard deviation analysis). (B) Selected genes of interest associated with cell survival. Plots are log_2(FC) with 95% confidence intervals (gray shaded area). Red indicates upregulation, blue indicates downregulation. (C) Significantly differentially expressed genes associated with cell cycle progression over the time course of the experiment (≥l6σl at one or more time point, by standard deviation analysi). (D, E) Biological functions associated with cell cycle progression are nearly universally significantly upregulated at the 10 and 24 hr time points. *p<0.05, **p<0.01, ***p<0.001, ****p<0.0001

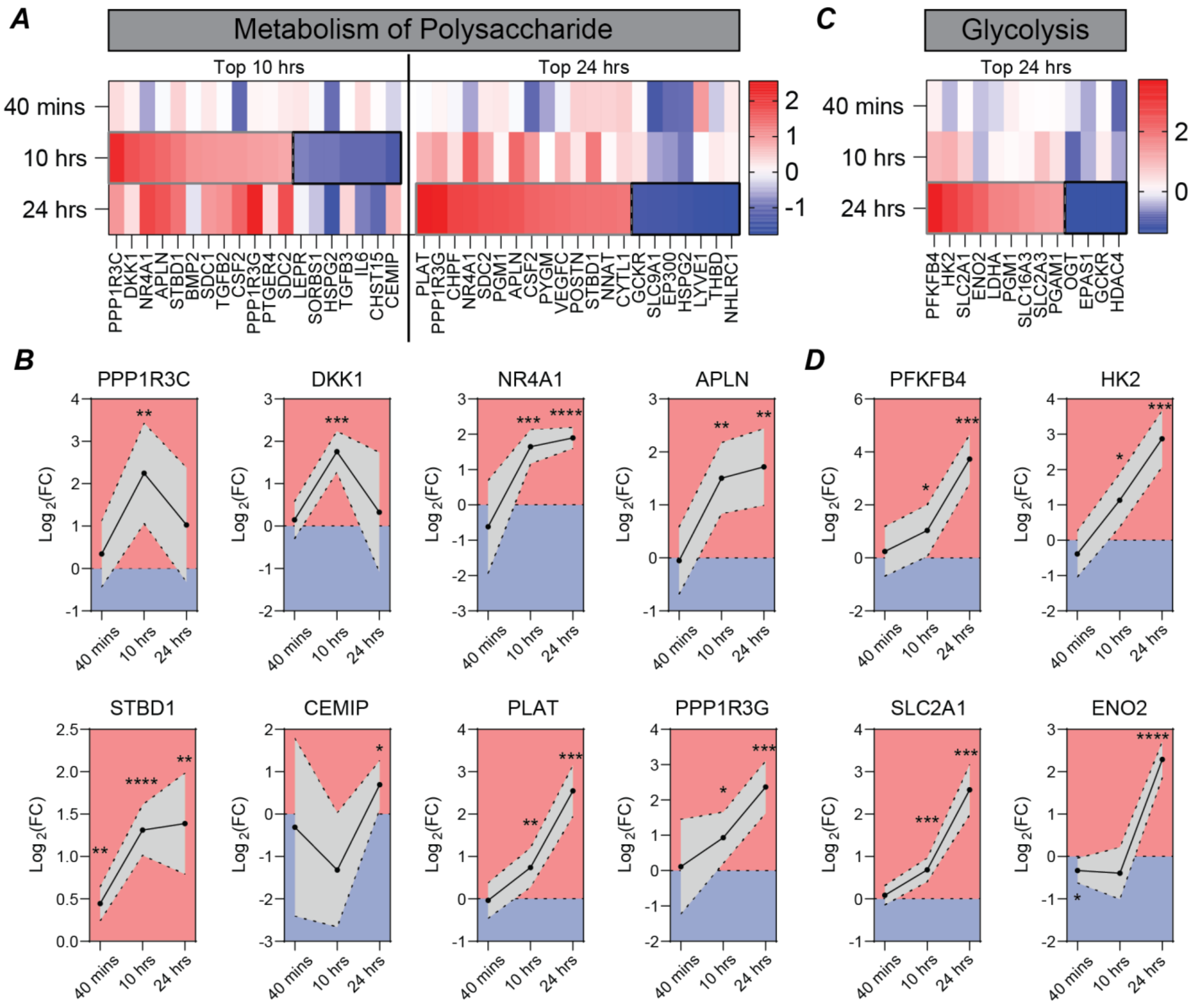

Figure S18. Differential expression of genes associated with metabolism of polysaccharide and glycolysis. (A) Significantly differentially expressed genes associated with metabolism of polysaccharide over the time course of the experiment (≥l3σl at one or more time point, by standard deviation analysis). (B) Highly differentially expressed genes (l6σl at one or more time point, by standard deviation analysis). Plots are $\log_2$(FC) with 95% confidence intervals (gray shaded area). Red indicates upregulation, blue indicates downregulation. (C) Significantly differentially expressed genes associated with glycolysis over the time course of the experiment (≥l3σl at one or more time point). (D) Highly differentially expressed genes (l6σl at one or more time point, by standard deviation analysis). Plots are $\log_2$(FC) with 95% confidence intervals (gray shaded area). Red indicates upregulation, blue indicates downregulation.*p<0.05, **p<0.01, ***p<0.001, ****p<0.0001.
